# Sesquiterpenoids and 2-(2-phenylethyl)chromones respectively acting as α-glucosidase and tyrosinase inhibitors from agarwood of an *Aquilaria* plant

**DOI:** 10.1080/14756366.2019.1576657

**Published:** 2019-04-22

**Authors:** Li Yang, Yi-Ling Yang, Wen-Hua Dong, Wei Li, Pei Wang, Xue Cao, Jing-Zhe Yuan, Hui-Qin Chen, Wen-Li Mei, Hao-Fu Dai

**Affiliations:** a Key Laboratory of Biology and Genetic Resources of Tropical Crops, Ministry of Agriculture, Institute of Tropical Bioscience and Biotechnology, Chinese Academy of Tropical Agricultural Sciences, Haikou, People's Republic of China;; b Hainan Key Laboratory for Research and Development of Natural Products from Li Folk Medicine, Haikou, People's Republic of China;; c Hainan Engineering Research Center of Agarwood, Haikou, People's Republic of China

**Keywords:** Agarwood, aquilaria plant, sesquiterpenoid, 2-(2-phenylethyl)chromone, α-glucosidase inhibition, tyrosinase inhibition

## Abstract

The ethyl ether extract of agarwood from an *Aquilaria* plant afforded six new sesquiterpenoids, Agarozizanol A − F (**1**−**6**), together with four known sesquiterpenoids and six known 2-(2-phenylethyl)chromones. Their structures were elucidated via detailed spectroscopic analysis, X-ray diffraction, and comparisons with the published data. All the isolates were evaluated for the α-glucosidase and tyrosinase inhibitory activities *in vitro*. Compounds **5**, **7**, **8**, and **10** showed significant inhibition of α-glucosidase with IC_50_ values ranging between 112.3 ± 4.5 and 524.5 ± 2.7 µM (acarbose, 743. 4 ± 3.3 µM). Compounds **13** and **14** exhibited tyrosinase inhibitory effect with IC_50_ values of 89.0 ± 1.7 and 51.5 ± 0.6 µM, respectively (kojic acid, 46.1 ± 1.3). In the kinetic studies, compounds **5** and **14** were found to be uncompetitive inhibitors for α-glucosidase and mixed type inhibitors for tyrosinase, respectively. Furthermore, molecular docking simulations revealed the binding sites and interactions of the most active compounds with α-glucosidase and tyrosinase.

## Introduction

Type 2 diabetes mellitus (T2DM), a chronic metabolic disorder disease, can cause life-threatening long-term complications including heart disease, strokes, blindness, kidney failure, and poor blood flow in the limbs^1^. In 2015, an estimated 1.6 million deaths directly resulted from diabetes, which is among the top 10 causes of death worldwide^2^. α-Glucosidase (EC 3.2.1.20) is an exo-acting enzyme, which implicates in the glycoprotein processing and carbohydrate metabolism. Additionally, α-glucosidase catalyzes the cleavage of *α*-1,4 glycosidic bonds from the nonreducing ends of oligosaccharides and provides high amount of intestine absorbable glucose, which results in the postprandial hyperglycaemia and complications associated with T2DM^3^ ^,^ ^4^. Therefore, α-glucosidase inhibition is a very effective therapy to delay glucose absorption and lower blood glucose level after food intake, which can potentially postpone the progression of T2DM^5^.

**Figure 1.**
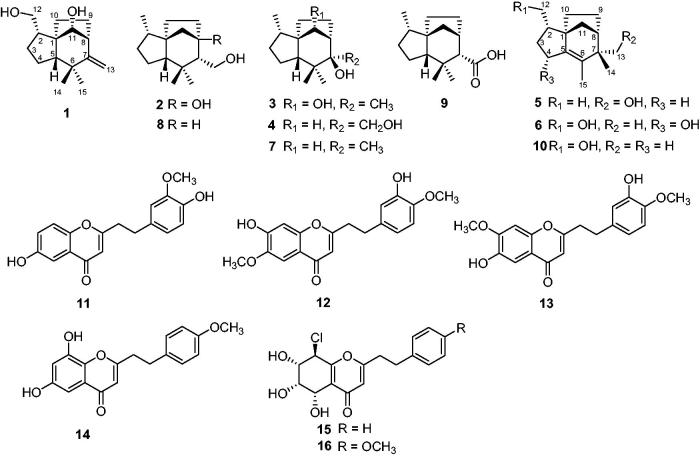
Structures of **1**–**16**.

As a precious traditional Chinese medicine, agarwood has been used for the treatment of joint pain, inflammatory related dailments, and diarrhoea, as well as a stimulant, sedative, and cardioprotective agent^6–8^. It is also popular as an incense for cultural and religious ceremonies and its essential oil is further processed into perfume in cosmetics. Previous chemical studies revealed that sesquiterpenoids and 2-(2-phenylethyl)chromone derivatives are the two principal and aroma components in agarwood. To date, over 130 sesquiterpenoids and 120 2-(2-phenylethyl)chromone derivatives have been isolated from agarwood^9^ and many of them exhibited various pharmacological properties including antibacterial^10^, anti-inflammatory^11^, α-glucosidase inhibitory^12^, acetylcholinesterase inhibitory^13^, cytotoxic^12^, neuroprotective^14^, and antidepressant activities^15^. As part of our long-term project to chemically and biologically characterize chemical constituents from agarwood, the ethyl ether extract of agarwood from an *Aquilaria* plant was found to potently inhibit α-glucosidase. Then, a phytochemical investigation on the agarwood of an *Aquilaria* plant was performed and 10 α-glucosidase inhibitory sesquiterpenoids including 6 new sesquiterpenoids, Agarozizanol A − F (**1**−**6**), together with 4 known sesquiterpenoids (Figure 1) were isolated and identified. Additionally, 6 known 2-(2-phenylethyl)chromones were also obtained. 2-(2-Phenylethyl)chromones are structurally similar to the flavones except for presence of additional ethyl group and many flavones show intriguing tyrosinase inhibitory activity^16–18^. Tyrosinase, a rate-limiting enzyme for the melanin biosynthesis, involves in pigmentation of skin^19^, browning in fresh vegetables and fruits^20^, cuticle formation in insects^21^ ^,^ ^22^, and neurodegeneration associated with Parkinson’s disease^23^. Hence, tyrosinase inhibitors have broad application in medicines, food preservatives, bio-insecticides, and cosmetic products. Inspired by this, All the 2-(2-phenylethyl)chromones were assayed for their tyrosinase inhibitory activity. Moreover, the inhibition mechanism were investigated. Herein, we report the isolation, structure elucidation, α-glucosidase and tyrosinase inhibitory activities, as well as kinetic and molecular docking studies of these compounds.

## Materials and methods

### General experimental procedures

Optical rotations were measured on an Anton Paar MCP 5100 polarimeter. IR spectra were obtained using a Nicolet 380 FT-IR instrument (Thermo, USA) using KBr pellets. HRESIMS were determined by a Waters Autospec Premier (Waters, USA) mass spectrometer. The NMR spectra were recorded on Bruker Avance 500 NMR spectrometers (Bruker, Germany) with TMS as an internal standard. The high performance liquid chromatography (HPLC) was performed with an analytic reversed-phased column (YMC–packed C_18_, 250 mm × 10 mm, 5 µm) (YMC, Japan) using a G1311C 1260 Quat Pump VL and detected with a G1315D 1260 DAD VL detector (190–500 nm) (Agilent Technologies 1260 infinity, USA). For column chromatography, silica gel (60–80, 200–300 mesh, Qingdao Haiyang Chemical Co., Ltd, China), ODS gel (20–45 µm, Fuji Silysia Chemical Co., Ltd, USA), and Sephadex LH-20 (Merck, Germany) were used. TLC analysis was performed on precoated silica gel GF254 plates (Qingdao Haiyang Chemical Co., Ltd, China), and spots were visualized by spraying with 5% H_2_SO_4_ in EtOH followed by heating. Thermo fisher scientific plate reader was used for enzyme inhibition assays.

### Plant material

The agarwood was purchased from Bangkok, Thailand in August 2014 and its original plant was identified as a species of the genus *Aquilaria* by gene sequence analysis of the ITS region. A voucher specimen (201408SLLK) was retained at the Institute of Tropical Bioscience and Biotechnology, Chinese Academy of Tropical Agricultural Sciences.

### Extraction and isolation

Air-dried agarwood (384.0 g) was ground and extracted with ethyl ether (1.5 L) for three times. The obtained extract was tested for the α-glucosidase inhibitory activity and showed inhibition rate of 44.13 ± 1.92% at concentration of 50 µg/mL (acarbose, 62.06 ± 4.77%). Then, the ethyl ether extract (20.4 g) was subjected to ODS column chromatography (CC) eluting with MeOH/H_2_O (v/v, 2:3, 1:1, 3:2, 7:3, 4:1, 9:1, 1:0) to obtain 16 fractions (Fr.1–Fr.16). Fr.4 (0.88 g) was separated by silica gel CC (petroleum ether/EtOAc, 50:1–5:2) to afford 12 subfrctions (Fr.4.1–Fr.4.12), Fr.4.5 was purified by silica gel CC (petroleum ether/EtOAc, 50:4) to yield compound **6** (3.5 mg). Purification of subfraction Fr.4.6 using silica gel CC (petroleum ether/EtOAc, 50:4) furnished compound **10** (3.3 mg). Fr.5 (5.3 g) was applied to silica gel CC eluted by petroleum ether/EtOAc (1:0–5:1), yielding 15 subfrctions (Fr.5.1–Fr.5.15). Separation of Fr.5.2 by silica gel CC (petroleum ether/CHCl_3_/EtOAc, 50:10:1) afforded compounds **1** (2.9 mg) and **5** (3.2 mg). Compounds **2** (2.9 mg) and **3** (2.8 mg) were obtained by separation of subfraction Fr.5.3 with silica gel CC (petroleum ether/EtOAc/isopropyl alcohol, 100:10:1). Subfraction Fr.5.4 was separated by silica gel CC (petroleum ether/CHCl_3_/isopropyl alcohol, 100:10:1) and preparative TLC, yielding compounds **9** (2.2 mg) and **4** (1.2 mg). Subfraction Fr.5.7 was chromatographed by silica gel CC (petroleum ether/CHCl_3_/isopropyl alcohol, 100:15:1) to obtain compounds **7** (1.2 mg) and **8** (2.3 mg). Fr.6 (0.6 g) was fractioned by silica gel CC (petroleum ether/CHCl_3_/CH_3_OH, 2:1:1), leading to isolation of 7 subfractions (Fr.6.1–Fr.6.7). Isolation of subfraction Fr.6.6 using silica gel CC and semi-preparative HPLC obtained compounds **15** (18.0 mg) and **16** (5.4 mg). Fr.7 (1.3 g) was divided into 13 subfractions (Fr.7.1–Fr.7.13) by silica gel CC using petroleum ether/EtOAc (500:1–0:1) as eluent. Subfraction Fr.7.11 was isolated via silica gel CC and semi-preparative HPLC to yield compounds **12** (2.2 mg) and **13** (3.0 mg). Fr.8 (0.8 g) was purified by silica gel CC (petroleum ether/EtOAc, 200:1-0:1), affording compounds **11** (0.8 mg) and **14** (3.5 mg).

### Agarozizanol A (1)

Colorless crystals; [*α*]25_D_ -3 (*c* 0.28, MeOH); IR (KBr) *ν*_max_ cm^−1^: 3252, 2956, 2870, 1629, 1462, 1090, and 1044; ^1^H and 13C NMR data, see Tables 1 and 2; HRESIMS *m/z* 259.1667 [M + Na]^+^ (calcd for C_15_H_24_NaO_2_, 259.1669); 1 D and 2 D NMR, IR spectra, and HRESIMS, see Figure S2–S9 in Supplementary material.

**Table 1.** ^1^H NMR (500 MHz) data for compounds **1**−**6**.

position	**1**^a^	**2**^a^	**3**^a^	**4**^b^	**5**^a^	**6**^a^
2	2.20, m	1.76, m	2.12, m	1.73, m	1.77, m	1.94, m
3a	1.84, m	1.85, m	1.88, m	1.76, m	1.80, m	2.38, dd (12.5,7.1)
3b	1.22, m	1.14, m	1.14, m	1.04, m	1.25, m	1.36, m
4a	1.70, m	1.53, m	1.59, m	1.42, m	2.24, dd (17.2, 9.1)	4.29, t (7.3)
4b			1.50, m		2.11, m	
5	1.66, m	1.54, m	1.74, m	1.64, dd (11.7, 8.5)		
7		1.80, m				
8	2.72, d (7.0)		1.87, m	2.18, dd (6.6, 4.7)	2.13, dd (7.5, 5.6)	1.83, m
9a	2.18, m	2.00, m	1.92, m	1.53, m	1.81, m	1.83, m
9b	1.65, m	1.56, m	1.60, m		1.63, m	1.66, m
10a	1.74, m	1.55, m	1.68, m	1.15, m	1.42, m	1.67, m
10b	1.53, m	1.26, m	1.32, m		1.18, m	1.51, m
11a	3.34, m	1.89, d (10.1)	4.01, brs	1.54, d (10.4)	1.57, dd (10.5, 5.5)	1.49, t (7.7)
11b		1.09, dt (10.1, 2.2)		1.25, dd (10.4, 4.8)	1.32, m	1.30, m
12a	3.53, t (10.7)	0.87, d (6.7)	1.01, d (6.9)	0.81, d (6.1)	0.95, d (6.5)	3.70, dd (10.6, 6.1)
12b	3.42, dd (10.7, 4.5)					3.55 dd (10.6,7.7)
13a	4.81, d (1.5)	3.89, dd (11.1, 9.4)	1.19, s	3.40, d (4.5)	3.60, d (10.9)	1.06, s
13b	4.79, d (1.5)	3.77, dd (11.1, 2.7)			3.54 d (10.9)	
14	1.13, s	1.06, s	0.87, s	0.85, s	1.07, s	1.03, s
15	1.10, s	0.79, s	0.95, s	0.80, s	1.41, s	1.60, s

^a^Recorded in CD_3_OD, ^b^Recorded in DMSO-d_6_.

**Table 2.** ^13^C NMR (125 MHz) data for compounds **1**−**6**.

position	**1**^a^	**2**^a^	**3**^a^	**4**^b^	**5**^a^	**6**^a^
1	58.4, C	53.0, C	57.0, C	51.9, C	54.0, C	52.3, C
2	47.5, CH	40.2, CH	39.3, CH	38.7, CH	41.1, CH	44.7, CH
3	25.8, CH_2_	31.5, CH_2_	33.0, CH_2_	30.5, CH_2_	33.5, CH_2_	35.9, CH_2_
4	22.3, CH_2_	21.9, CH_2_	23.3, CH_2_	20.7, CH_2_	28.4, CH_2_	81.3, CH
5	59.1, CH	60.4, CH	56.7, CH	55.6, CH	146.7, C	143.1, C
6	37.4, C	35.8, C	40.2, C	37.4, C	126.1, C	137.0, C
7	162.6, C	58.1, CH	80.3, C	76.1, C	46.5, C	41.9, C
8	55.4, CH	83.6, C	58.4, CH	43.0, CH	43.0, CH	48.0, CH
9	27.9, CH_2_	21.8, CH_2_	24.4, CH_2_	25.5, CH_2_	25.1, CH_2_	25.3, CH_2_
10	20.2, CH_2_	32.2, CH_2_	20.4, CH_2_	21.0, CH_2_	29.7, CH_2_	39.5, CH_2_
11	84.3, CH	52.2, CH_2_	83.5, CH	37.4, CH	37.8, CH_2_	30.9, CH_2_
12	62.6, CH_2_	14.4, CH_3_	18.4, CH_3_	19.1, CH_3_	14.4, CH_3_	64.3, CH_2_
13	108.2, CH_2_	62.4, CH_2_	23.9, CH_3_	62.7, CH_2_	66.7, CH_2_	28.6, CH_3_
14	27.9, CH_3_	33.9, CH_3_	27.6, CH_3_,	27.8, CH_3_	23.3, CH_3_	25.4, CH_3_
15	32.1, CH_3_	17.8, CH_3_	21.8, CH_3_	14.5, CH_3_	13.3, CH_3_	12.8, CH_3_

^a^Recorded in CD_3_OD, ^b^Recorded in DMSO-d_6_.

### Agarozizanol B (2)

Colorless crystals; [*α*]25_D_ +15 (*c* 0.28, MeOH); IR (KBr) *ν*_max_ cm^−1^: 3413, 2956, 2870, 1450, 1381, 1261, and 1059; ^1^H and 13C NMR data, see Tables 1 and 2; HRESIMS *m*/*z* 261.1820 [M + Na]^+^ (calcd for C_15_H_26_NaO_2_, 261.1825). 1 D and 2 D NMR, IR spectra, and HRESIMS, see Figures S10–S17 in Supplementary material.

### Agarozizanol C (3)

White amorphous powder; [*α*]25_D_ -11 (*c* 0.25, MeOH); IR (KBr) *ν*_max_ cm^−1^: 3444, 2956, 2868, 1456, 1376, 1250, 1091, and 1027; ^1^H and 13C NMR data, see Tables 1 and 2; HRESIMS *m*/*z* 261.1817 [M + Na]^+^ (calcd for C_15_H_26_NaO_2_, 261.1825). 1 D and 2 D NMR, IR spectra, and HRESIMS, see Figure S18-Figure S25 in Supplementary material.

### Agarozizanol D (4)

White amorphous powder; [*α*]25_D_ +14 (*c* 0.10, MeOH); IR (KBr) *ν*_max_ cm^−1^: 3439, 2950, 2867, 1457, 1374, and 1067; ^1^H and 13C NMR data, see Table 1 and 2; HRESIMS *m*/*z* 261.1826 [M + Na]^+^ (calcd for C_15_H_26_NaO_2_, 261.1825). 1 D and 2 D NMR, IR spectra, and HRESIMS, see Figure S26–S33 in Supplementary material.

### Agarozizanol E (5)

White amorphous powder; [*α*]25_D_ -7 (*c* 0.33, MeOH); IR (KBr) *ν*_max_ cm^−1^: 3447, 2938, 2863, 1627, 1456, 1377, 1260, 1094, and 1034; ^1^H and 13C NMR data, see Tables 1 and 2; HRESIMS *m*/*z* 243.1707 [M + Na]^+^ (calcd for C_15_H_24_NaO, 243.1719). 1 D and 2 D NMR, IR spectra, and HRESIMS, see Figure S34–S41 in Supplementary material.

### Agarozizanol F (6)

White amorphous powder; [*α*]25_D_ +2 (*c* 0.36, MeOH); IR (KBr) *ν*_max_ cm^−1^: 3446, 2928, 2867, 1617, 1454, 1382, and 1084; ^1^H and 13C NMR data, see Tables 1 and 2; HRESIMS *m*/*z* 259.1662 [M + Na]^+^ (calcd for C_15_H_24_NaO_2_, 259.1669). 1 D and 2 D NMR, IR spectra, and HRESIMS, see Figure S42–S49 in Supplementary material.

### X-ray crystallographic analysis of compound 2

A suitable crystal of **2** obtained from MeOH was selected and determined on a Bruker D8 QUEST diffractometer at 296.15 K. Crystal data for compound **2**: C_15_H_26_O_2_ (*M* = 238.36 g/mol), orthorhombic, space group *P2_1_2_1_2_1_*, *a* = 6.7545(7) Å, *b* = 8.8069(8) Å, *c* = 23.886(2) Å, *V* = 1420.9 (2) Å^3^, *Z* = 4, *T* = 296(2) K, µ (Cu K*α*)=0.556 mm^−1^, *Dcalc* = 1.114 g/cm^3^, 9320 reflections collected (7.402° ≤ 2θ ≤ 136.888°), 2598 unique (*Rint* = 0.1110, *Rsigma* = 0.0937). The final *R*1 was 0.0559 (I > 2σ(I)) and *wR2* was 0.1624 (all data), Flack parameter = 0.3(8). The crystallographic data for **2** have been deposited at the Cambridge Crystallographic Data Centre under the reference number CCDC 1873716. This data can be obtained free of charge via http://www.ccdc.cam.ac.uk/conts/retrieving.html or from CCDC, 12 Union Road, Cambridge, CB21EZ, UK; (fax: +44-(0)1223-336033 or e-mail: deposit@ccdc.cam.ac.uk).

### α-Glucosidase inhibitory assay

The α-glucosidase (G5003, Sigma-Aldrich) was obtained from *Saccharomyces cerevisiae* and the enzyme inhibitory assay was performed using formerly described method^24^ with some modifications. The samples were dissolved in DMSO and 2-fold diluted to afford a serial concentrations (the highest final concentration was set at 750 µM because of low solubility of **1**–**10** in buffer). 10 µL sample was incubated with 100 µL α-glucosidase solution (0.2 U/mL in 100 mM phosphate buffer (pH: 6.8)) at 37 °C for 15 min. Then, 40 µL of 2.5 mM *p*-nitrophenyl-α-d-glucopyranoside (*p*-NPG) were added and further incubated at 37 °C for 15 min. DMSO instead of compound was used as control and the blank wells contained buffer in place of substrate. The OD values were measured at 405 nm with microplate reader. Acarbose was used as reference compound. The percentage inhibition was calculated using the following equation:

$$\%\;\text{inhibition}=\left[1–\left({\text{OD}}_{\text{compound}}–{\text{OD}}_{\text{blank}}\right)/\left({\text{OD}}_{\text{control}}–{\text{OD}}_{\text{blank}}\right)\right]\times 100$$

### Tyrosinase inhibitory assay

All the isolated compounds (**1**–**16**) were tested for the inhibitory activity against tyrosinase (T3824, Sigma-Aldrich) from mushroom according to the previously reported method^16^ with slight modifications. Briefly, 130 µL of tyrosinase (50 U/mL) solvated in 50 mM phosphate buffer (pH: 6.8) were mixed with 20 µL of 2-fold serial dilutions (from 1 mM to 0.03125 mM) of compounds in DMSO, and transferred into a 96-well plate. After 5 min pre-incubation of the mixture at 37 °C, 50 µL of 2 mM l-tyrosine in buffer were added and incubated for additional 20 min at 37 °C. The control wells contained DMSO instead of compound, and the blank wells were added with buffer in place of l-tyrosine. Kojic acid was used as a positive control. The absorbance was measured at 495 nm using microplate reader. The percentage inhibition was calculated using the formula as below:

$$\%\;\text{inhibition}=\left[1–\left({\text{OD}}_{\text{compound}}–{\text{OD}}_{\text{blank}}\right)/\left({\text{OD}}_{\text{control}}–{\text{OD}}_{\text{blank}}\right)\right]\times 100$$

### Kinetic analysis

To determine the mechanisms by which the most effective compound **5** inhibited α-glucosidase and compound **14** inhibited tyrosinase, the enzyme kinetic analysis was investigated at various concentrations of substrate (2.5, 1.25, 0.625, and 0.3125 mM *p*-NPG for α-glucosidase; 4.0, 2.0, 1.0, and 0.5 mM tyrosine for tyrosinase) in the absence or presence of different test inhibitor concentrations (0, 187.5, and 750 µM of **5**; 0, 25, and 100 µM of **14**) according to the inhibition assay described above. The data were expressed as double-reciprocal Lineweaver − Burk plot and the inhibition constants (K_I_ and K_IS_) were obtained from secondary plots of slope and y intercept of lines from Lineweaver-Burk plot against inhibitor^25^.

### Molecular docking study

The X-ray structures of mushroom tyrosinase (PDB: 2Y9X) at a resolution of 2.78 Å and α-glucosidase (PDB: 3A4A) co-crystalized with glucose at a resolution of 1.6 Å were achieved from RCSB Protein Data Bank (www.rcsb.org). All hydrogen atoms and gasteiger charges in the protein structure were added by AutoDockTools and molecular docking simulations were performed with Autodock 4.2 software using the Lamarckian Genetic Algorithm^26^ ^,^ ^27^. Compounds **5** and **10** were docked into the catalytic site of α-glucosidase with a grid box of 50 Å × 50 Å × 50 Å at 0.375 Å space, centered at coordinates of *x* = 21.275, *y* = −0.741, and *z* = 18.635. Genetic Algorithm parameters were set at Runs 50 and the maximum number of evals was set as medium (25000000). For the compounds **13** and **14,** mixed type tyrosinase inhibitors, a blind molecular docking simulation was performed (Runs 20 and the maximum number of evals was set as short (250000)). The size of grid box was set at 100 Å × 100 Å × 100 Å in the *x, y*, and *z* dimensions at 0.375 Å space. This computational docking identified the site that bound tightly to the ligand. Then, a refined docking simulation was conducted with smaller grid box of 30 Å × 44 Å × 30 Å at 0.375 Å space, centered at the above identified binding site (*x* = −16.345, *y* = −36.541, *z* = −22.916). The Genetic Algorithm parameters were set at Runs 50 and the maximum number of evals was set as medium (25000000). The predicted geometries were ranked by the binding energies and clustered at RMSD value of 2 Å, and the best pose was selected for further analysis.

## Results and discussion

### Structure elucidation

**Figure 2.**
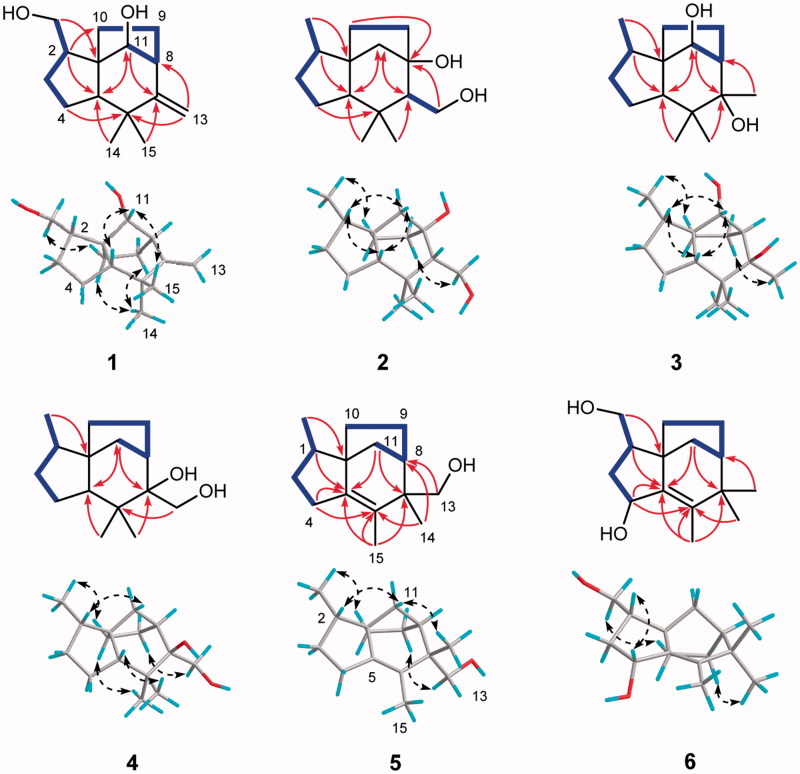
Key ^1^H-^1^H COSY (bold lines), HMBC (arrows), and ROESY (dashed double arrows) correlations of compounds **1−6**.

Compound **1** was obtained as colorless crystals and its molecular formula was assigned to be C_15_H_24_O_2_ based on the HRESIMS ion peak [M + Na]^+^ at *m*/*z* 259.1667 (calcd for C_15_H_24_NaO_2_, 259.1669), requiring 4 degrees of unsaturation. The ^1^H NMR data (Table 1) of **1** showed resonances for two methyl groups at *δ*_H_ 1.10 (3H, s) and 1.13 (3H, s), two exocyclic olefinic protons at *δ*_H_ 4.81 (1H, d, *J* = 1.5 Hz) and 4.79 (1H, d, *J* = 1.5 Hz), and one oxygenated methylene at *δ*_H_ 3.53 (1H, t, *J* = 10.7 Hz) and 3.42 (1H, dd, *J* = 10.7, 4.5 Hz). The ^13^C NMR data (Table 2) of **1** showed fifteen carbon signals which were categorized by HSQC spectrum as two methyl groups, six methylenes (one oxygenated), four methines, and three quaternary carbons. Apart from the double bond (*δ*_C_ 162.6, 108.2), the remaining three degrees of unsaturation combined with above mentioned spectroscopic data implied that **1** was a tricyclic sesquiterpenoid. In the ^1^H-^1^H COSY spectrum (Figure 2), two spin systems were observed: H_2_-12–H-2–H_2_-3–H_2_-4 and H-11–H-8–H_2_-9–H_2_-10. The two spin systems in association with key HMBC correlations from H_2_-12 to C-1, from H_2_-4 to C-1, C-5, and C-6, from H-5 to C-6, C-7, C-1, C-10, and C-11, from H_2_-13 to C-6, C-7, and C-8, and from H_3_-14 and H_3_-15 to C-5, C-6, and C-7 constructed the planar structure of **1**, possessing a prezizaane skeleton, as shown in Figure 1.

The relative configuration of **1** was clarified unambiguously from analysis of the ROESY spectrum. The ROE cross peak of H_2_-12 with H-10a revealed that H_2_-12 and H-10a located at the same side and assumed that they were *α* oriented. H-11 showed ROESY correlations with H-5 and H_3_-14, indicating they were cofacial and assigned to be *β* orientation. Thus, the structure of **1** was determined to be as shown in Figure 1, named Agarozizanol A.

Compound **2** was isolated as colorless crystals and had a molecular formula C_15_H_26_O_2_ on the basis of its HRESIMS and 13C NMR spectroscopic data. The ^1^H NMR data (Table 1) of **2** revealed the presence of three methyl group at *δ*_H_ 0.87 (3H, d, *J* = 6.7 Hz), 1.06 (3H, s), and 0.79 (3H, s) and one oxygenated methylene at *δ*_H_ 3.89 (1H, dd, *J* = 11.1, 9.4 Hz) and 3.77 (1H, d, *J* = 11.1, 2.7 Hz). The ^13^C NMR (Table 2) and HSQC spectra of **1** showed the resonances for three methyl groups, six methylenes (one oxygenated), three methines, and three quaternary carbons (one oxygenated). The aforementioned spectroscopic data were similar to those of **8**, a co-occurring known prezizaane sesquiterpenoids Jinkohol II^28^. The only difference was the presence of additional oxidized quaternary carbon signal (*δ*_C_ 83.6) and the absence of a methine signal in the NMR spectra of **2**. The HMBC correlations (Figure 2) of H_2_-13, H_2_-11, and H_2_-10 with this carbon suggested that the oxygenated carbon was C-8. H_3_-12 showed ROESY correlations with H-10a, suggesting they were cofacial. Other ROESY interactions of H-2 with H-5, H-11b and H_3_-14 with H-5 and H-7 were found, revealing that these protons were resided on the same face. Therefore, the structure of **2** was deduced as Agarozizanol B shown in Figure. 1.

Compound **3**, a white amorphous powder, was found to have a molecular formula of C_15_H_26_O_2_ from the ion peak [M + Na]^+^ at *m*/*z* 261.1817 (calcd for C_15_H_26_NaO_2_, 261.1825) in the HRESIMS. The ^1^H and ^13^C NMR data (Table 1 and 2) of **3** exhibited the characteristic signals for prezizane sesquiterpenoid including four methyls, four methylenes, four methines, and three quaternary carbons. The NMR spectroscopic data of **3** closely resemble that of **7**, Jinkohol I^29^, with exception of deshieled C-11 at *δ*_C_ 83.5. This chemical shift indicated the attachment of a hydroxyl to C-11, which was confirmed by HMBC correlation from H-11 to C-9/C-10/C-5/C-7 and ^1^H-^1^H COSY correlations of H-11–H-8–H-9–H_2_-10 (Figure 2). The ROESY correlations of H-2/H-11/H-5 suggested that these protons were cofacial and *β* oriented. Additional ROESY cross peaks of H_3_-12/H-10a and H_3_-13/H-9b permitted assignment of *α* orientation to H_3_-12 and H_3_-13. Summarizing these data, the structure of **3** was determined as shown in Figure 1 and named Agarozizanol C.

Obtained as white amorphous powder, compound **4** showed an ion peak [M + Na]^+^ at *m/z* 261.1826 (calcd for C_15_H_26_NaO_2_, 261.1825) in the HRESIMS, consistent with a molecular formula of C_15_H_26_O_2_. The structure of **4** was similar to that of known compound **7**, except for the presence of additional hydroxyl at C-13 in **4**, which was verified by the deshielded chemical shift of C-13 at *δ*_C_ 62.7 and the molecular formula of **4** with one more oxygen atom than that of **7**. The ROESY interactions of H_3_-12 with H_2_-10, H_2_-9 with H_2_-13 and H_3_-15 (Figure 2), indicated these protons located at the same side of the molecule and were assigned to be *α* oriented. While H-5, H_3_-14, H-2, and H-11_a_ were oriented on the other side by their ROESY correlations. Hence, the structure of **4** was established as a new epimer of synthetic (+)-prezizaene diol^30^, named Agarozizanol D.

Compound **5** was obtained as white amorphous powder and was shown to have a molecular formula C_15_H_24_O based on its ion peak [M + Na]^+^ at *m*/*z* 243.1707 (calcd for C_15_H_24_NaO, 243.1719) in HRESIMS. Resonances of three methyls at *δ*_H_ 0.95 (3H, d, *J* = 6.5 Hz), 1.07 (3H, s), and 1.41 (3H, s), and one oxygenated methylene at *δ*_H_ 3.60 (1H, d, *J* = 10.9 Hz) and 3.54 (1H, d, *J* = 10.9 Hz) were observed in the ^1^H NMR spectrum of compound **5.** The ^13^C NMR (Table 2) and HSQC spectra of **5** showed the presence of three methyl groups, six methylenes (one oxygenated), two methines, and four quaternary carbons including two olefinic carbons. These spectroscopic data of **5** were similar to those of compound **10** ^31^, a known zizaene sesquiterpenoid. The major difference was that the hydroxyl, which located at C-12 in the **10**, was attached to the C-13 in the compound **5**. This assignment was confirmed by the deshielded chemical shifts of C-13 at *δ*_C_ 66.7 and the HMBC correlations between H_2_-13 and C-6/C-14/C-7/C-8 (Figure 2). The ROESY correlations of H_3_-12/H-10a, H_2_-13/H-9a, and H_3_-14/H-11b were observed, allowing the assignment of *α* orientation for H_3_-12 and H-9a, and *β* orientation for H_3_-14 and H-11b. Based on all the above evidence, the structure of **5** was identified as shown in Figure 1 and named as Agarozizanol E.

Compound **6** was obtained as white amorphous powder and displayed an ion peak [M + Na]^+^ at *m*/*z* 259.1662 (calcd for C_15_H_24_NaO_2_, 259.1669) in its HRESIMS, corresponding to a molecular formula C_15_H_24_O_2_ with one more oxygen atom than that of **10**. The ^1^H and ^13^C NMR data of **6** were closely similar to those of **10** ^31^ with only difference being the deshieled chemical shift of CH-4 (*δ*_H_ 4.29, *δ*_C_ 81.3), which indicated the presence of hydroxyl at C-4. This deduction was further verified by HMBC correlations of H-4 with C-1/C-5/C-6 (Figure 2). The *α* orientation of CH_2_OH-12, OH-4, and CH_2_-10 was assigned from the ROESY correlations of H_2_-12/H-10b and H-2/H-4. Accordingly, the structure of **6** was characterized as shown in Figure.1, named Agarozizanol F.

**Figure 3.**
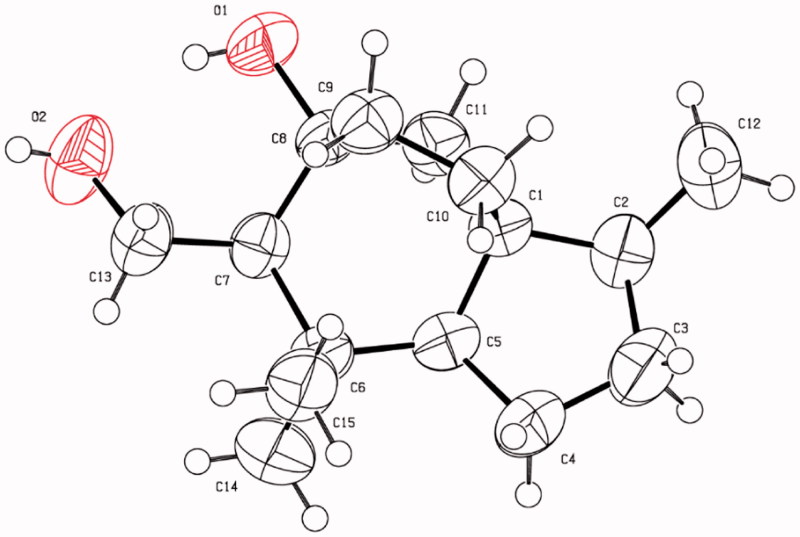
X-ray crystallographic structure of **2**.

On the basis of their structural similarity, compounds **1**−**6** should be generated from a common precursor in their biosynthetic pathway (Supplementary material, Figure S1), and possessed the same stereochemistry at C-1 and C-2. Fortunately, single-crystals of **2** were obtained and subjected to X-ray diffraction experiment with Cu K*α* radiation (Figure 3). However, the imperfect Flack parameter [0.3(8)] only allowed confirmation of the above deduced planar structure and relative configuration of **2**. Therefore, further effort was needed to determine the absolute configuration of **1−6**.

By comparing their experimental spectroscopic data with reported data in the literature, the known compounds were identified as Jinkohol I (**7**)^29^, Jinkohol II (**8**)^28^, Jinkoholic acid (**9**)^32^, isokhusenol (**10**)^31^, 6-hydroxy-2-[2-(3-methoxy-4-hydroxyphenyl)ethyl]chromone (**11**)^33^, 6-methoxy-7-hydroxy-2-[2-(3-hydroxy-4-methoxyphenyl)ethyl]chromone (**12)** ^34^, 6-hydroxy-7-methoxy-2-[2-(3-hydroxy-4-methoxyphenyl)ethyl]chromone (**13**)^14^, 6,8-dihydroxy-2-[2-(4-methoxyphenyl)ethyl]chromone (**14**)^12^, 8-chloro-2–(2-phenylethyl)-5,6,7-trihydroxy-5,6,7,8-tetrahydrochromone (**15**)^35^, 8-chloro-2–(2-(4-methoxyphenyl)ethyl)-5,6,7-trihydroxy-5,6,7,8-tetrahydrochromone (**16**)^36^.

### Bioactivity assays, kinetic analysis, and molecular docking simulations

#### α-Glucosidase inhibitory activity assay, kinetic analysis, and molecular docking study

All the isolated compounds were assessed for the inhibition against α-glucosidase. The results were listed in the Table 3. Compounds **5**, **7**, **8**, and **10** showed inhibitory effect on α-glucosidase (IC_50_ values ranging from 112.3 ± 4.5 to 524.5 ± 2.7 µM) superior to acarbose (IC_50_, 743. 4 ± 3.3 µM). Compounds **2**, **4**, and **9** also possessed inhibitory activity comparable to the positive control.

**Table 3.** α-Glucosidase inhibitory activities of compounds **1**−**16** ^a^.

Compound	α-glucosidase inhibitory activity^b^	
	750 μM (%)	IC_50_ ± SD (μM)
**1**	37.4 ± 1.4	N.T.
**2**	42.0 ± 2.7	N.T.
**4**	49.9 ± 1.8	N.T.
**5**	93.4 ± 3.1	112.3 ± 4.5
**7**	86.9 ± 0.4	407.0 ± 3.3
**8**	77.3 ± 1.0	524.5 ± 2.7
**9**	48.9 ± 1.1	N.T.
**10**	92.1 ± 3.4	218.8 ± 5.6
acarbose^c^	50.7 ± 1.2	743. 4 ± 3.3

^a^Compounds **3**, **6**, and **11**−**16** were inactive toward α-glucosidase at 750 μM (inhibition rate <30%). ^b^Values represent means ± SD based on three individual experiments. ^c^Positive control. N.T.: not tested.

**Figure 4.**
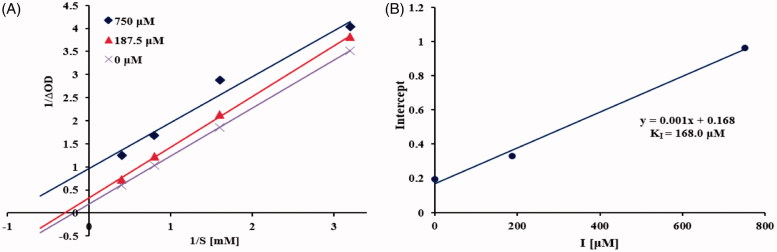
Kinetics of α-glucosidase inhibition by **5**. Lineweaver-Burk plot of **5** against α-glucosidase (A). Secondary plot of y intercept vs. inhibitor for determination of inhibition constant (B).

To elucidate the type of inhibition of isolated sesquiterpenoids with α-glucosidase, compound **5** with most potency was selected for the kinetic analysis. The Lineweaver-Burk plot (Figure 4(A)) of **5** was established and revealed that *K_m_* and *V_max_* decreased in a parallel fashion, indicating that **5** acted as an uncompetitive α-glucosidase inhibitor by binding only with the enzyme-substrate complex^37^. The inhibition constant was calculated using secondary plot of y intercept versus concentration of inhibitor, yielding K_I_ value of 168.0 µM (Figure 4(B)).

**Figure 5.**
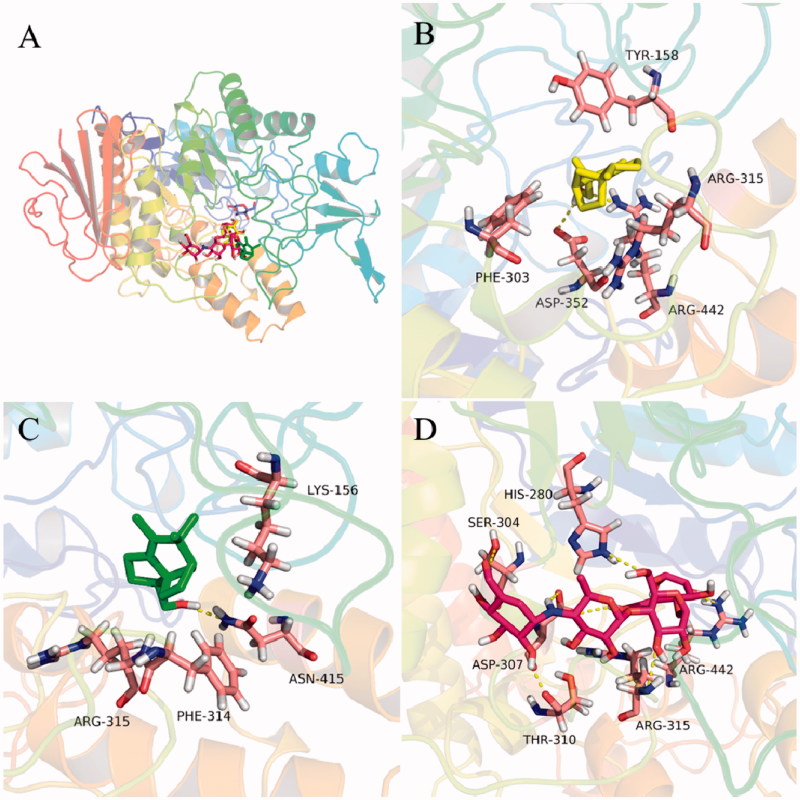
Docking analysis of **5** (yellow sticks), **10** (green sticks), and acarbose (red sticks) with α-glucosidase co-crystalized with glucose (cyan sticks). Binding mode of **5**, **10**, acarbose, and glucose in the active site of α-glucosidase (A). 3D cartoon diagram of the interactions of **5** (B), **10** (C), and acarbose (D) with α-glucosidase.

In order to rationalize the uncompetitive inhibition mechanism indicated by kinetic study, molecular modeling studies were performed. The most active compounds **5** and **10** were well docked into the catalytic site of the α-glucosidase (Figure. 5) with binding energies of −7.2 and −7.1 kcal/mol, respectively. The docking results revealed that compounds **5** and **10** bound to the entrance part of the active site cavity of α-glucosidase, therefore blocking the release of product (glucose) after the enzyme catalyzed reaction was completed^37^. This may explain why compound **5** behaved as an uncompetitive inhibitor of α-glucosidase. Specifically, compound **5** bound to the active site through hydrogen bonds with residues Asp 352 and Arg 442, and hydrophobic interactions with Tyr 158, Phe 303, and Arg 315 (Figure 5(B)). Compound **10** took hydrogen bonding with Asn 415 and hydrophobic interactions with Lys 156, Phe 314, and Arg 315 (Figure 5(C)). These interactions may provide hints for designing of a new class of α-glucosidase inhibitor.

#### Tyrosinase inhibitory actvity assay, kinetic analysis, and molecular docking study

2-(2-Phenylethyl)chromones **11**−**16** as well as sesquiterpenoids **1**−**10** were assayed for the inhibition toward the tyrosinase. As illustrated in Table 4, Compounds **14** and **13** displayed potent inhibitory activity against tyrosinase with IC_50_ values of 51.5 ± 0.6 and 89.0 ± 1.7 µM, respectively (kojic acid, 46.1 ± 1.3 µM). Compound **15** also exhibited weak inhibition effect with inhibition rate of 34.8 ± 1.4% at 100 µM. Whereas, compounds **1**−**12** and **16** were inactive to the tyrosinase up to concentration of 100 µM (inhibition rate <30%).

**Table 4.** Tyrosinase inhibitory activities of compounds **1**−**16** ^a^.

compound	tyrosinase inhibitory activity^b^	
	100 μM (%)	IC_50_ ± SD (μM)
**13**	53.4 ± 0.7	89.0 ± 1.7
**14**	65.0 ± 1.8	51.5 ± 0.6
**15**	34.8 ± 1.4	N.T.
kojic acid^c^	72.5 ± 0.5	46.1 ± 1.3

^a^Compounds **1**–**12**, and **16** were inactive against tyrosinase at 100 μM (inhibition rate < 30%). ^b^Values represent means ± SD based on three individual experiments. ^c^Positive control. N.T.: not tested.

**Figure 6.**
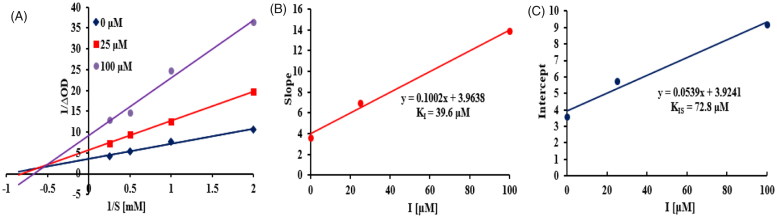
Kinetics of tyrosinase inhibition by **14**. Lineweaver-Burk plot of **14** against tyrosinase (A). Secondary re-plot of slope vs. inhibitor for determination of inhibition constant KI (B). Secondary re-plot of y intercept vs. inhibitor for determination of inhibition constant KIS (C).

As shown in the Lineweaver-Burk plot of the most active compound **14** (Figure 6(A)), increasing inhibitor concentration resulted in a decrease in *V_max_* and an increase in *K_m_*, which indicated a mixed type inhibition for **14** against tyrosinase^25^. Thus, compound **14** inhibited the tyrosinase not only by binding with the free enzyme but also with the enzyme − substrate complex. The inhibition constants of **14** binding with the free enzyme (*K_I_*) and with enzyme-substrate complex (*K_IS_*) were determined by the slope and the *y* intercept versus the inhibitor concentration, respectively. From Figure 6(B,C), the inhibition constants *K_I_* and *K_IS_* of **14** were calculated to be 39.6 µM and 72.8 µM, respectively, by secondary re-plots, indicating that compound **14** effectively bound to free enzyme as compared to enzyme-substrate complex.

**Figure 7.**
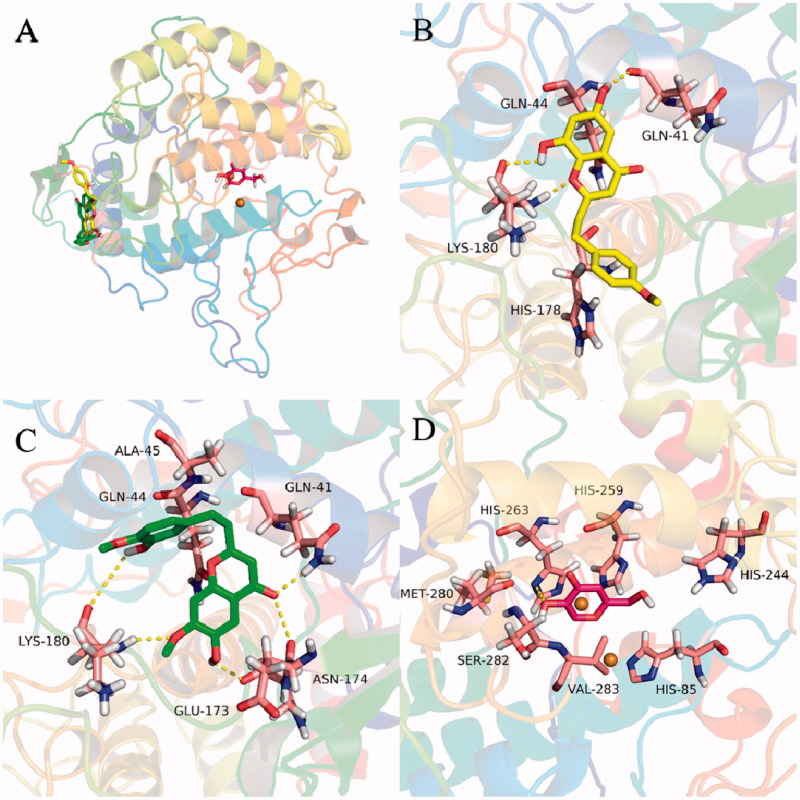
Docking analysis of **13** (green sticks), **14** (yellow sticks), and kojic acid (red sticks) with tyrosinase. Binding mode of **13**, **14**, and kojic acid with tyrosinase (A). 3D cartoon diagram of the interactions of **14** (B), **13** (C), kojic acid (D) with tyrosinase.

To further gain insight into the binding mechanism of 2-(2-phenylethyl)chromones with tyrosinase, molecular docking simulations were performed. As presented in Figure 7, compounds **13** and **14** were able to anchor the same binding pocket of tyrosinase with the binding energy of −7.49 and −7.75 kcal/mol, respectively. Compound **14** formed hydrogen bonds with residues Gln 41, Lys 180, and hydrophobic interactions with residues His 178 and Gln 44. However, compound **13** bound to the binding site with a different mode and interacted via hydrogen bonds with residues Gln 41, Lys 180, Asn 174, and Glu173, and hydrophobic interactions with residues Gln 44 and Ala 45. The differences may stem from the substitution of ring A and B of 2-(2-phenylethyl)chromones. These results unveiled a possible allosteric site of tyrosinase by 2-(2-phenylethyl)chromones and will be helpful for understanding the binding interactions between 2-(2-phenylethyl)chromones and tyrosinase.

## Conclusions

The ethyl ether extract of agarwood from an *Aquilaria* plant afforded 6 previously undescribed sesquiterpenoids, 4 known sesquiterpenoids, as well as 6 known 2-(2-phenylethyl)chromones. Among them, compounds **2**, **4**, **5**, and **7**–**10** showed intriguing inhibitory activity against α-glucosidase. The combined enzyme kinetic and molecular docking studies were performed and revealed that the most effective compound **5**, a new zizaane-type sesquiterpenoid, behaved as an uncompetitive α-glucosidase inhibitor and blocked catalytic reaction by interacting with the entrance of active site of α-glucosidase. Uncompetitive inhibitors are considered to be superior to competitive and noncompetitive inhibitors in drug development and are expected to display better *in vivo* efficacy^37^ ^,^ ^38^. Therefore, compound **5** could serve as a new promising lead compound for synthesis of more potent derivatives as α-glucosidase inhibitors. Additionally, compounds **13** and **14** manifested remarkable tyrosinase inhibitory effect. Compound **14** with the most potency was revealed to have affinity for the allosteric site of tyrosinase as a mixed type inhibitor. Agarwood formation is due to a self-defense mechanism initiated by the tree to fight off pests and pathogens invading through holes and wounds on the tree surface^6^. 2-(2-Phenylethyl)chromone is one of the two main components of agarwood and our study revealed the 2-(2-phenylethyl)chromones exert inhibitory activity on tyrosinase, which may provide the chemical evidence for the self-defense mechanism of the agar-producing tree against pests.

## Supplementary Material

Supplemental Material
