# Supplementary material for: Sesquiterpenoids and 2-(2-phenylethyl)chromones respectively acting as α-glucosidase and tyrosinase inhibitors from agarwood of an Aquilaria plant
Source: J Enzyme Inhib Med Chem. 2019 Apr 22;34(1):853–62. doi: 10.1080/14756366.2019.1576657 (PMC6495113; doi:10.1080/14756366.2019.1576657)
Supplement: Supplemental Material [file IENZ_A_1576657_SM5758.pdf]

Sesquiterpenoids and 2-(2-phenylethyl)chromones respectively acting as α-glucosidase and tyrosinase inhibitors from agarwood of an *Aquilaria* plant

Li Yang[a,b], Yi-Ling Yang[a,b], Wen-Hua Dong[a,b,c], Wei Li[a,b,c], Pei Wang[a,b,c], Xue Cao[a,b], Jing-Zhe Yuan[a,b,c], Hui-Qin Chen[a,b,c], Wen-Li Mei[a,b,c], Hao-Fu Dai[a,b,c,*]

[a] *Key Laboratory of Biology and Genetic Resources of Tropical Crops, Ministry of Agriculture, Institute of Tropical Bioscience and Biotechnology, Chinese Academy of Tropical Agricultural Sciences, Haikou, 571101, People's Republic of China.*

[b] *Hainan Key Laboratory for Research and Development of Natural Products from Li Folk Medicine, Haikou, 571101, People's Republic of China.*

[c] *Hainan Engineering Research Center of Agarwood, Haikou, 571101, People's Republic of China.*

---

*Corresponding author: daihaofu@itbb.org.cn (H.-F. Dai).

Supplementary material

Contents

1. Plausible biogenetic pathway for compounds 1-6......4

Figure S1. Plausible biogenetic pathway for compounds **1-6**......4

2. 1D and 2D NMR spectra, IR spectra, and HRESIMS of compounds 1-6......5

Figure S2. ^{1}H NMR (500 MHz) spectrum of compound **1** in CD_3OD......5

Figure S3. ^{13}C NMR (125 MHz) spectrum of compound **1** in CD_3OD......5

Figure S4. HSQC spectrum of compound **1** in CD_3OD......6

Figure S5. ^{1}H-^{1}H COSY spectrum of compound **1** in CD_3OD......6

Figure S6. HMBC spectrum of compound **1** in CD_3OD......7

Figure S7. ROESY spectrum of compound **1** in CD_3OD......7

Figure S8. IR spectrum of compound **1**......8

Figure S9. HRESIMS of compound **1**......8

Figure S10. ^{1}H NMR (500 MHz) spectrum of compound **2** in CD_3OD......9

Figure S11. ^{13}C NMR (125 MHz) spectrum of compound **2** in CD_3OD......9

Figure S12. HSQC spectrum of compound **2** in CD_3OD......10

Figure S13. ^{1}H-^{1}H COSY spectrum of compound **2** in CD_3OD......10

Figure S14. HMBC spectrum of compound **2** in CD_3OD......11

Figure S15. ROESY spectrum of compound **2** in CD_3OD......11

Figure S16. IR spectrum of compound **2**......12

Figure S17. HRESIMS of compound **2**......12

Figure S18. ^{1}H NMR (500 MHz) spectrum of compound **3** in CD_3OD......13

Figure S19. ^{13}C NMR (125 MHz) spectrum of compound **3** in CD_3OD......13

Figure S20. HSQC spectrum of compound **3** in CD_3OD......14

Figure S21. ^{1}H-^{1}H COSY spectrum of compound **3** in CD_3OD......14

Figure S22. HMBC spectrum of compound **3** in CD_3OD......15

Figure S23. ROESY spectrum of compound **3** in CD_3OD......15

Figure S24. IR spectrum of compound **3**......16

Figure S25. HRESIMS of compound **3**......16

Figure S26. ^{1}H NMR (500 MHz) spectrum of compound **4** in DMSO-d_6 17
Figure S27. ^{13}C NMR (125 MHz) spectrum of compound **4** in DMSO-d_6 17
Figure S28. HSQC spectrum of compound **4** in DMSO-d_6 18
Figure S29. ^{1}H-^{1}H COSY spectrum of compound **4** in DMSO-d_6 18
Figure S30. HMBC spectrum of compound **4** in DMSO-d_6 19
Figure S31. ROESY spectrum of compound **4** in DMSO-d_6 19
Figure S32. IR spectrum of compound **4** 20
Figure S33. HRESIMS of compound **4** 20
Figure S34. ^{1}H NMR (500 MHz) spectrum of compound **5** in CD_3OD 21
Figure S35. ^{13}C NMR (125 MHz) spectrum of compound **5** in CD_3OD 21
Figure S36. HSQC spectrum of compound **5** in CD_3OD 22
Figure S37. ^{1}H-^{1}H COSY spectrum of compound **5** in CD_3OD 22
Figure S38. HMBC spectrum of compound **5** in CD_3OD 23
Figure S39. ROESY spectrum of compound **5** in CD_3OD 23
Figure S40. IR spectrum of compound **5** 24
Figure S41. HRESIMS of compound **5** 24
Figure S42. ^{1}H NMR (500 MHz) spectrum of compound **6** in CD_3OD 25
Figure S43. ^{13}C NMR (125 MHz) spectrum of compound **6** in CD_3OD 25
Figure S44. HSQC spectrum of compound **6** in CD_3OD 26
Figure S45. ^{1}H-^{1}H COSY spectrum of compound **6** in CD_3OD 26
Figure S46. HMBC spectrum of compound **6** in CD_3OD 27
Figure S47. ROESY spectrum of compound **6** in CD_3OD 27
Figure S48. IR spectrum of compound **6** 28
Figure S49. HRESIMS of compound **6** 28

1. Plausible biogenetic pathway for compounds 1-6

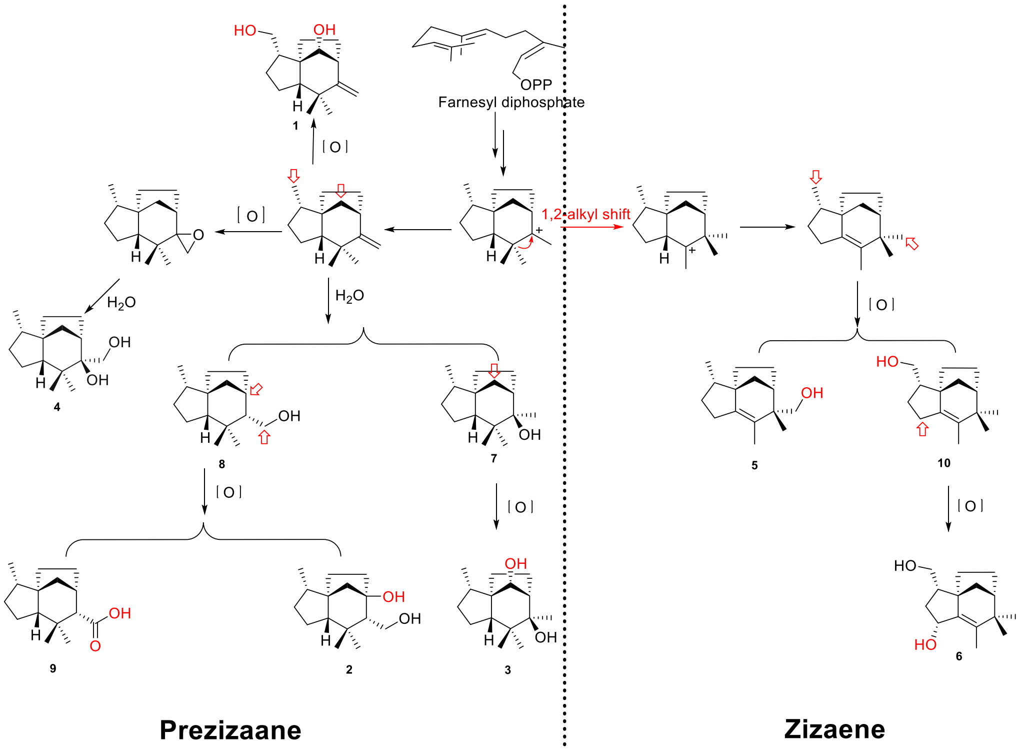

Figure S1. Plausible biogenetic pathway for compounds **1-6**

1 Lin X, Hopson R, Cane DE. Genome mining in *Streptomyces coelicolor*: molecular cloning and characterization of a new sesquiterpene synthase. J Am Chem Soc 2006;128:6022-6023.

2 Lin X, Cane DE. Biosynthesis of the sesquiterpene antibiotic albaflavenone in *Streptomyces coelicolor*. Mechanism and stereochemistry of the enzymatic formation of epi-isozizaene, J Am Chem Soc 2009;131:6332-6333.

3 Aaron JA, Lin X, Cane DE, et al. Structure of epi-isozizaene synthase from *Streptomyces coelicolor* A3(2), a platform for new terpenoid cyclization templates, Biochemistry 2010;49:1787-1797.

2. 1D and 2D NMR spectra, IR spectra, and HRESIMS of compounds 1-6

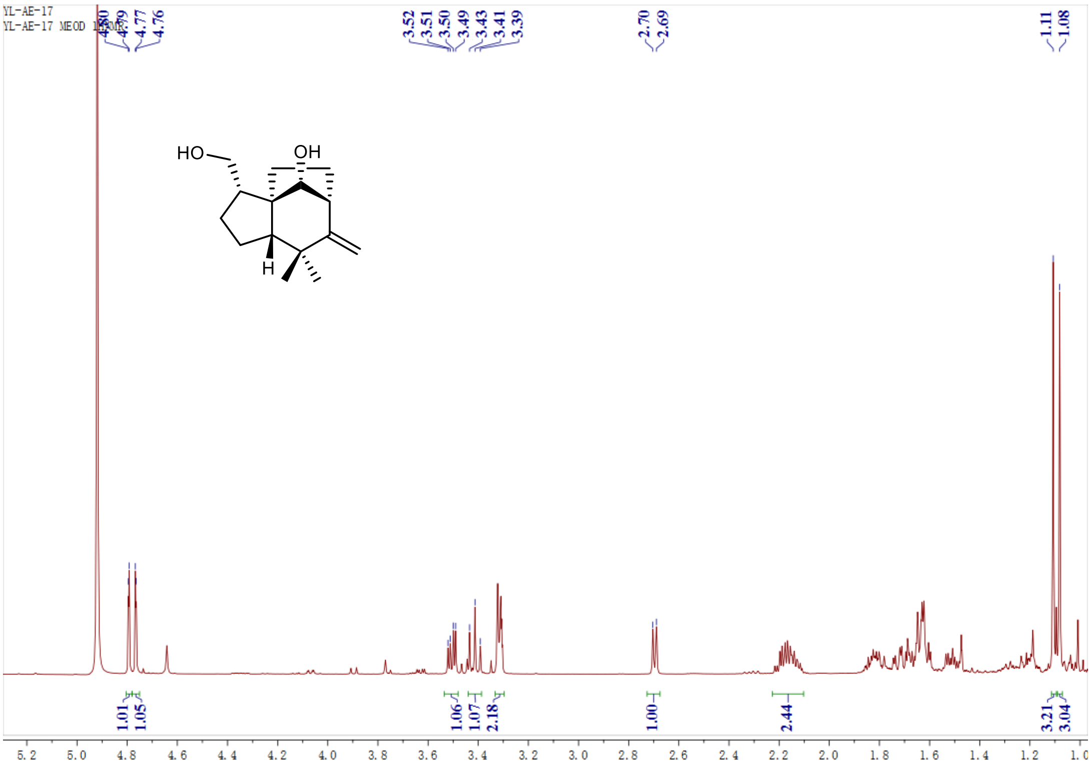

Figure S2. ^{1}H NMR (500 MHz) spectrum of compound **1** in CD_3OD

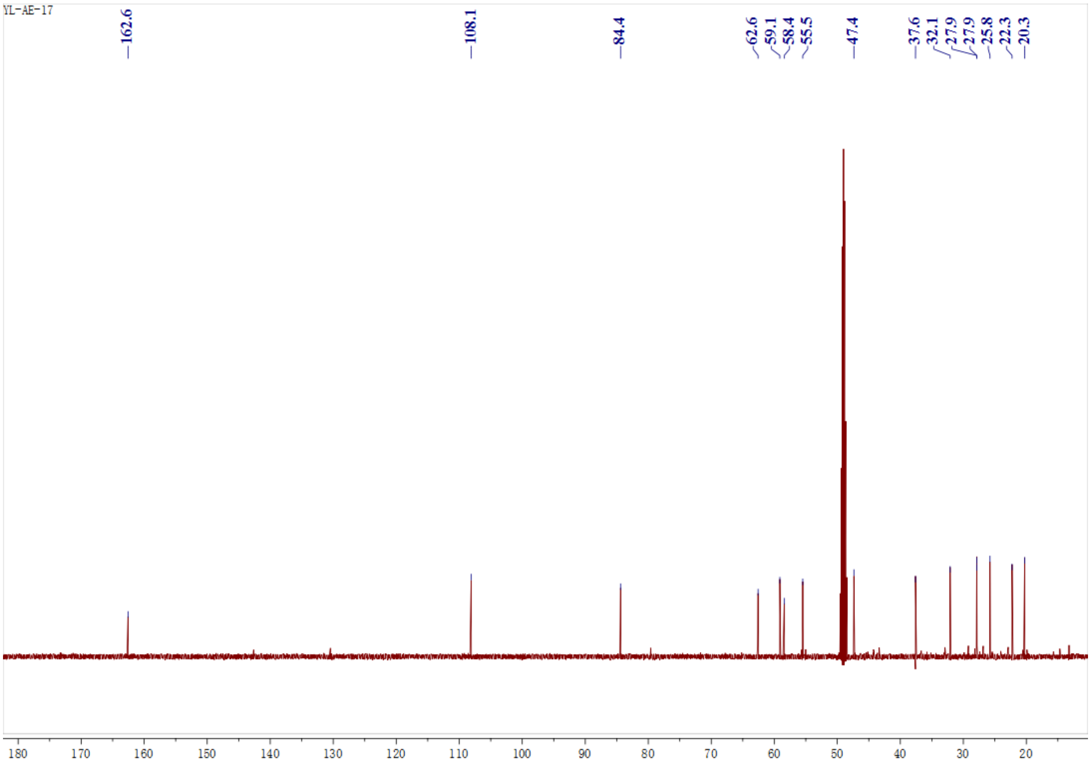

Figure S3. ^{13}C NMR (125 MHz) spectrum of compound **1** in CD_3OD

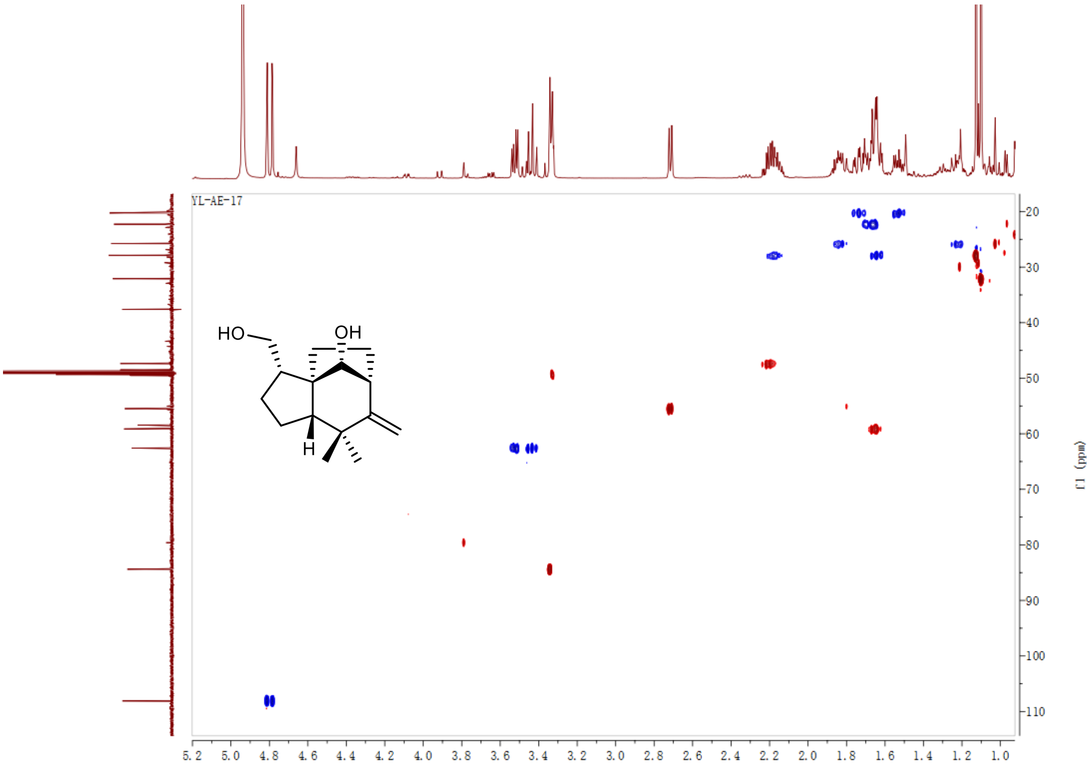

Figure S4. HSQC spectrum of compound **1** in CD_3OD

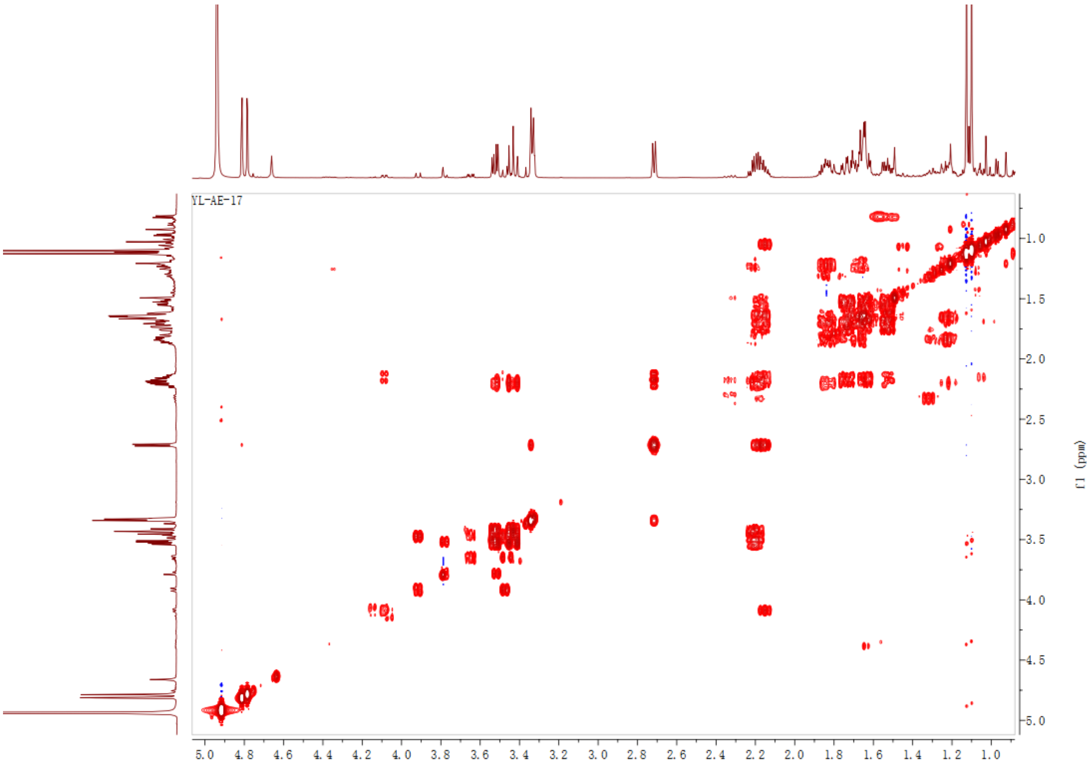

Figure S5. ^{1}H-^{1}H COSY spectrum of compound **1** in CD_3OD

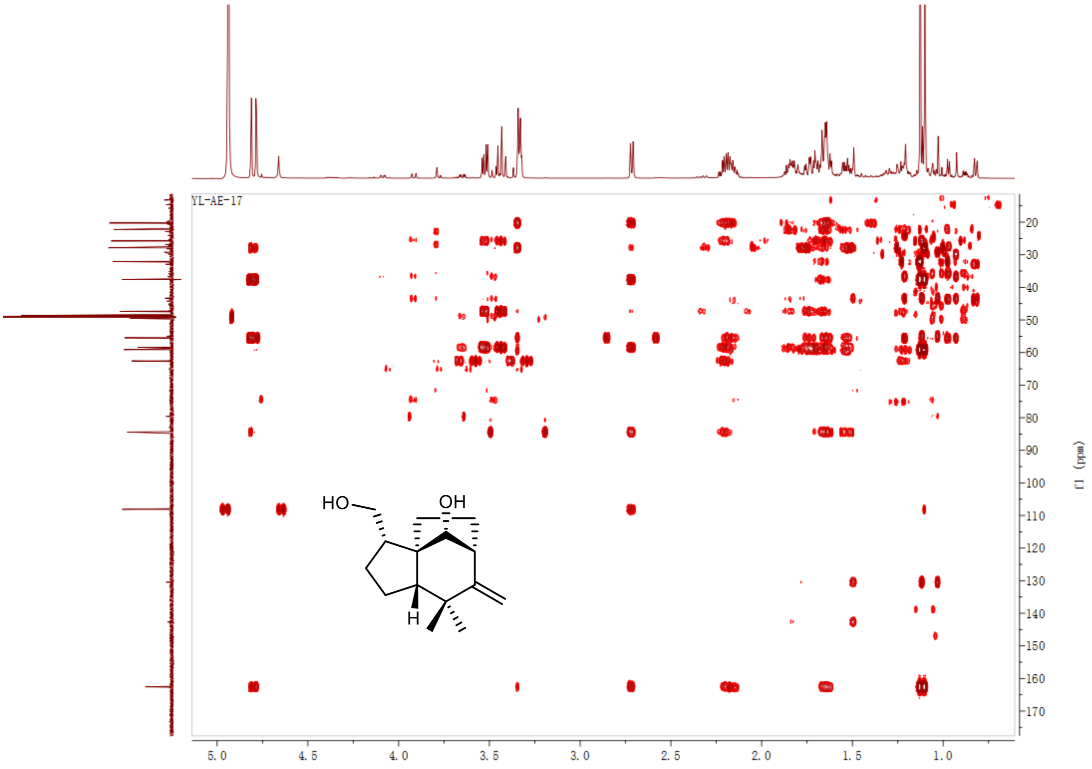

Figure S6. HMBC spectrum of compound **1** in CD_3OD

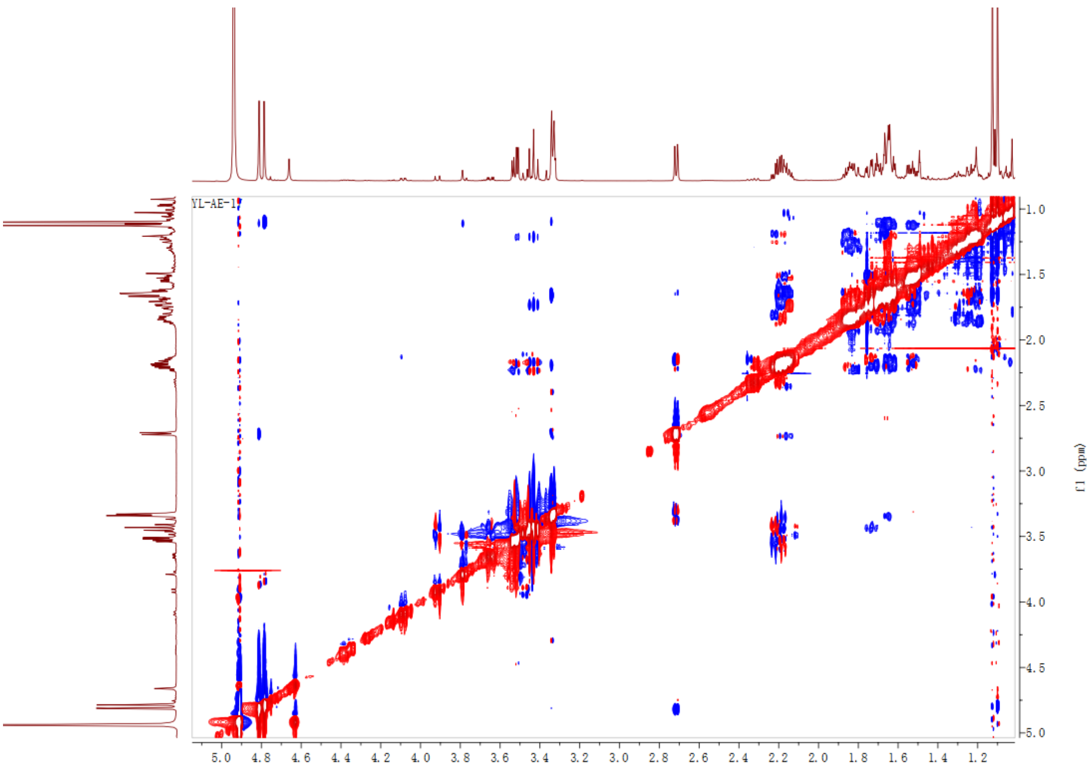

Figure S7. ROESY spectrum of compound **1** in CD_3OD

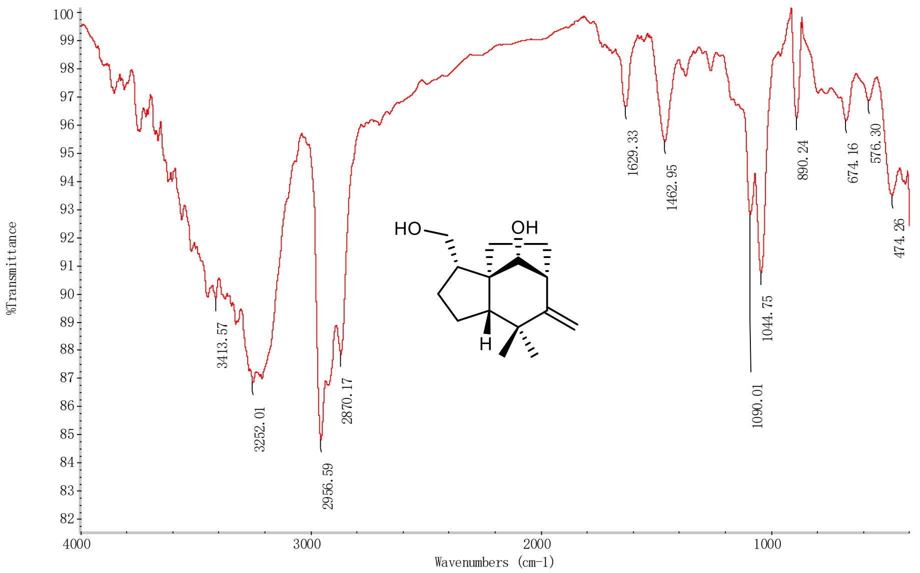

Figure S8. IR spectrum of compound **1**

Mass Spectrum SmartFormula Report

Analysis Info

Analysis Name	D:\Data\A501\YJZ\2018.10.10\E-17.d	Acquisition Date	2018-10-10 16:50:46
Method	DirectInfusion - MS - positive.m	Operator	Demo User
Sample Name	E-17	Instrument	compact 8255754.20156
Comment			

Acquisition Parameter

Source Type	ESI	Ion Polarity	Positive	Set Nebulizer	0.4 Bar
Focus	Not active	Set Capillary	4500 V	Set Dry Heater	180 °C
Scan Begin	50 m/z	Set End Plate Offset	-500 V	Set Dry Gas	4.0 l/min
Scan End	1300 m/z	Set Charging Voltage	2000 V	Set Divert Valve	Source
		Set Corona	0 nA	Set APCI Heater	0 °C

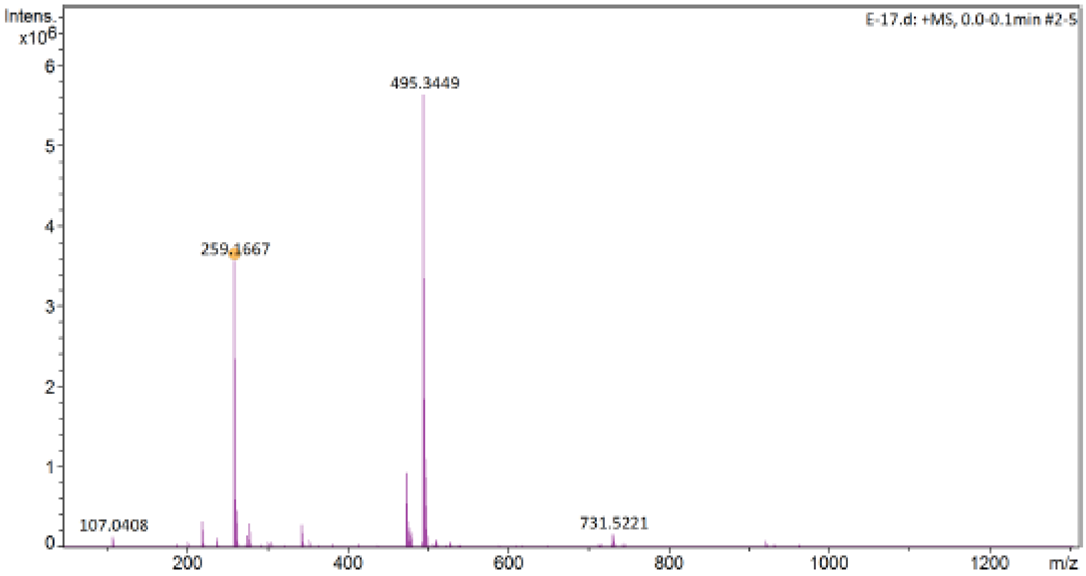

Sum Formula	Ion Formula	Meas. m/z	m/z	err [mDa]	err [ppm]
C15H24O2	C15H24NaO2	259.1667	259.1669	0.2	0.7

Figure S9. HRESIMS of compound **1**

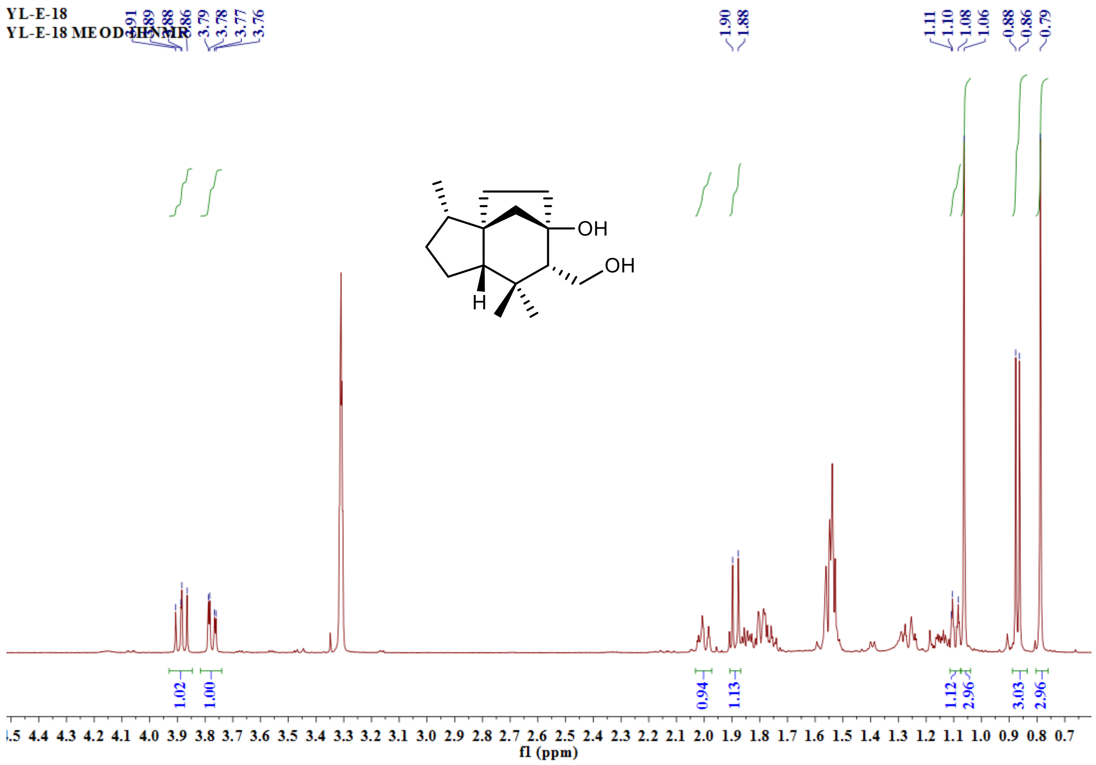

Figure S10. ^{1}H NMR (500 MHz) spectrum of compound **2** in CD_3OD

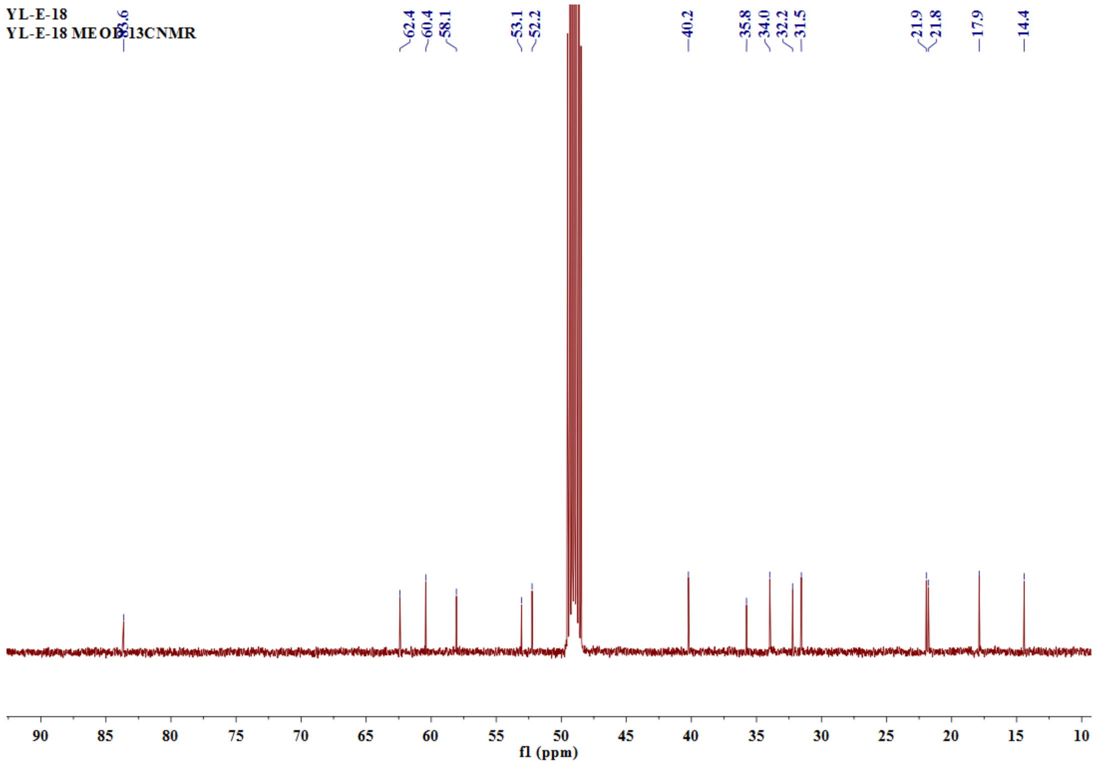

Figure S11. ^{13}C NMR (125 MHz) spectrum of compound **2** in CD_3OD

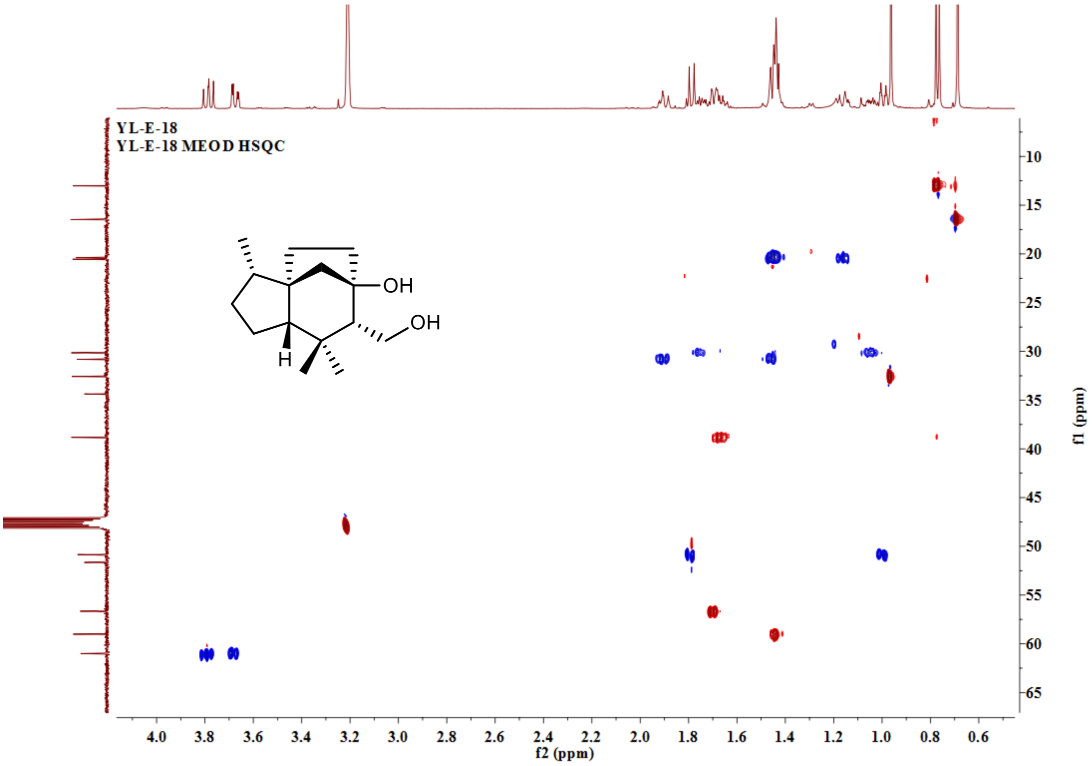

Figure S12. HSQC spectrum of compound **2** in CD_3OD

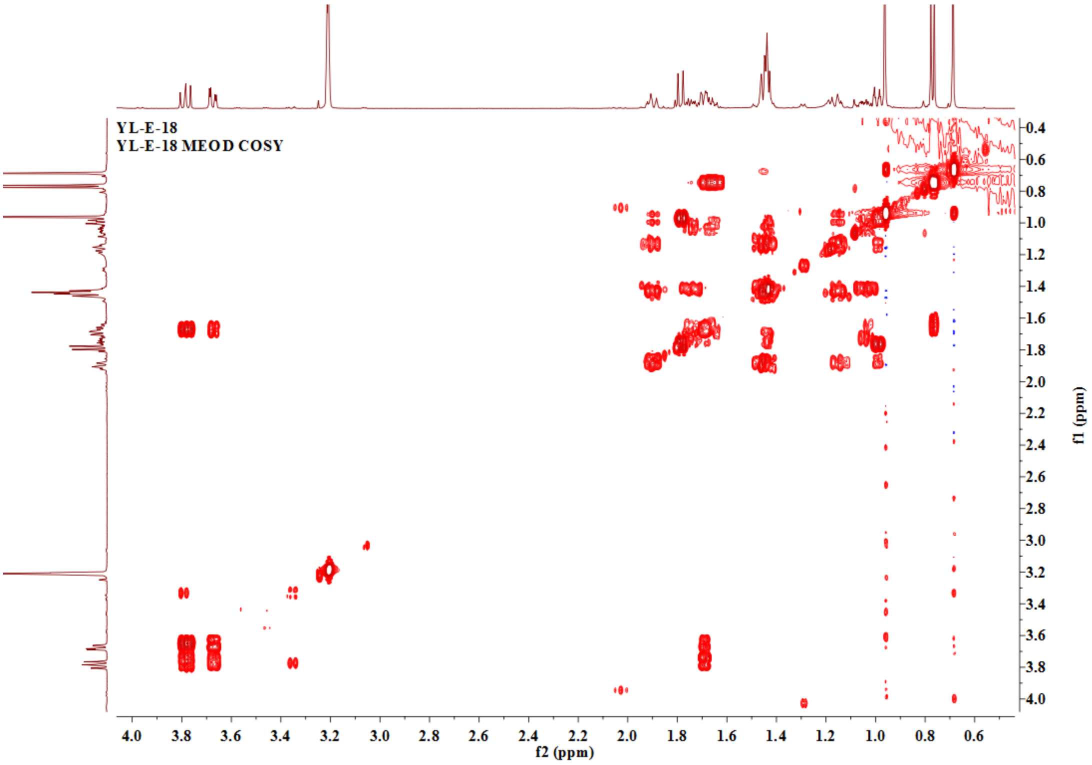

Figure S13. ^1H-^1H COSY spectrum of compound **2** in CD_3OD

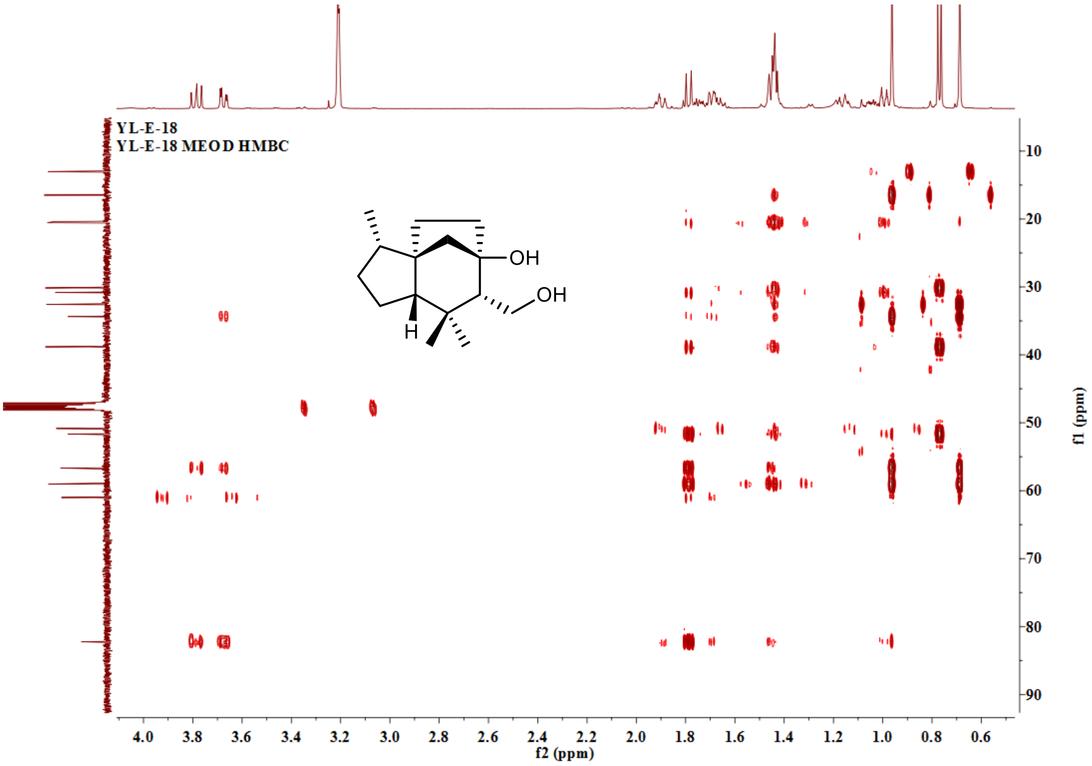

Figure S14. HMBC spectrum of compound **2** in CD_3OD

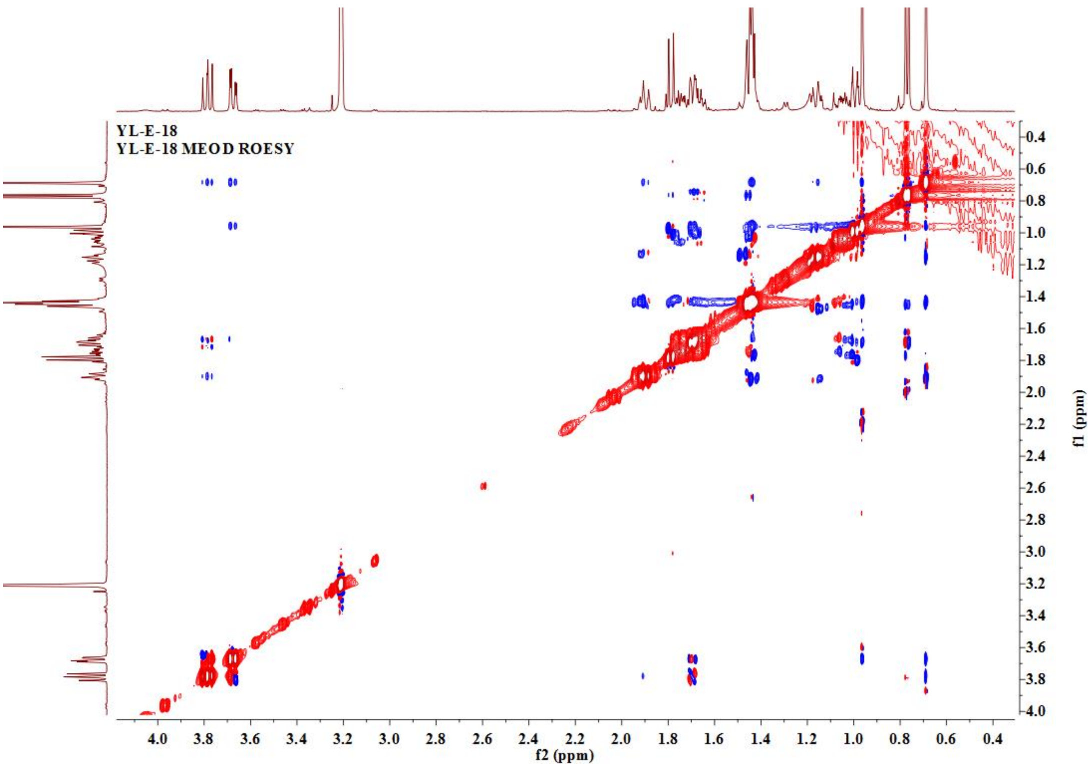

Figure S15. ROESY spectrum of compound **2** in CD_3OD

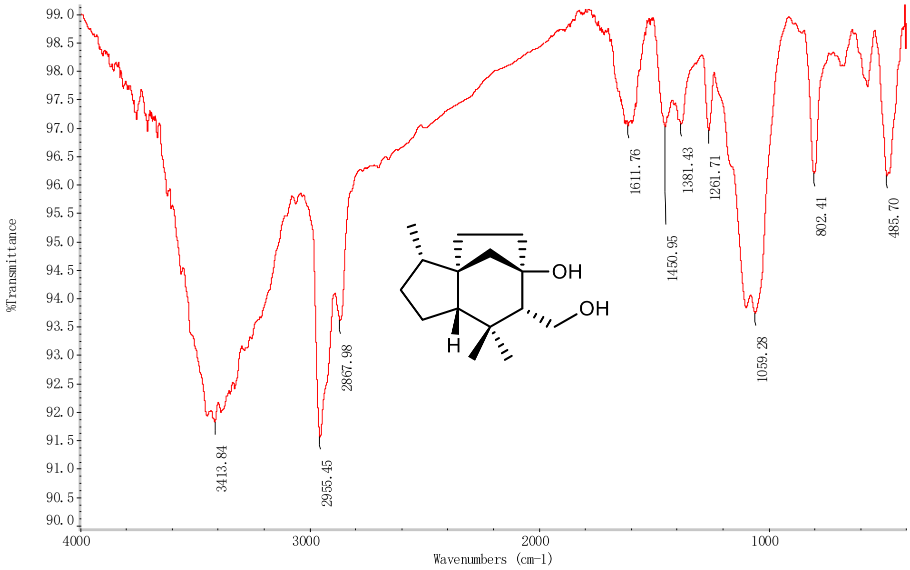

Figure S16. IR spectrum of compound **2**

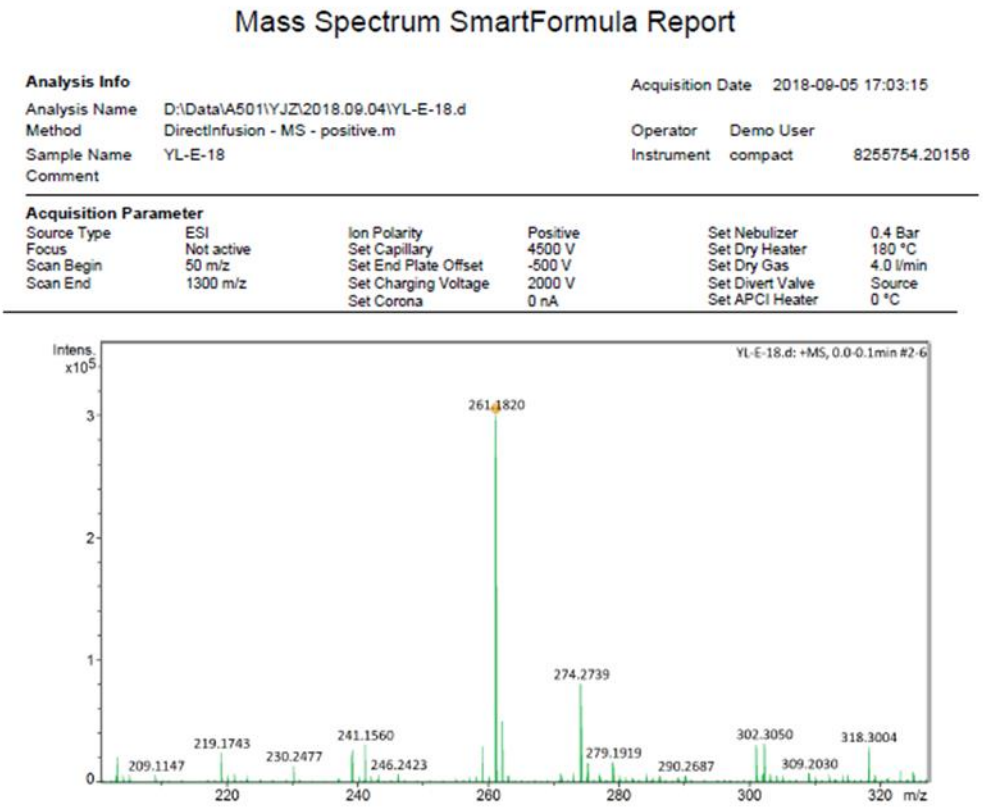

Sum Formula	Ion Formula	Meas. m/z	m/z	err [mDa]	err [ppm]
C15H26O2	C15H26NaO2	261.1820	261.1825	0.5	1.8

Figure S17. HRESIMS of compound **2**

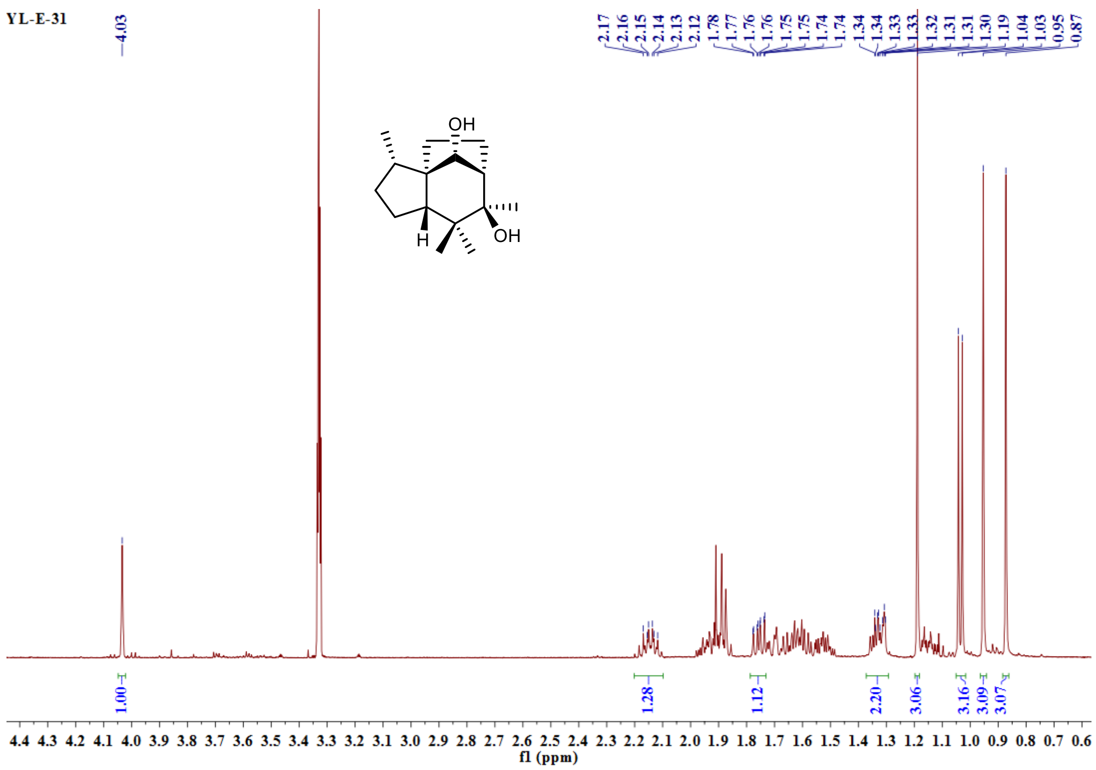

Figure S18. ^{1}H NMR (500 MHz) spectrum of compound **3** in CD_3OD

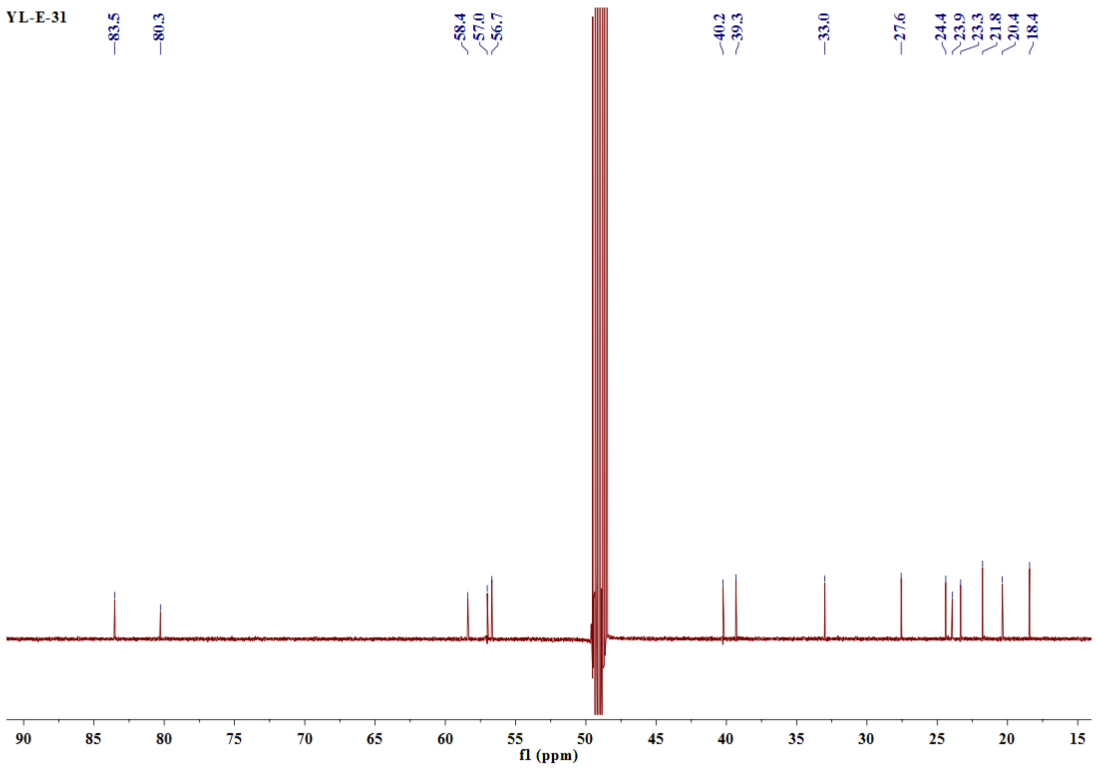

Figure S19. ^{13}C NMR (125 MHz) spectrum of compound **3** in CD_3OD

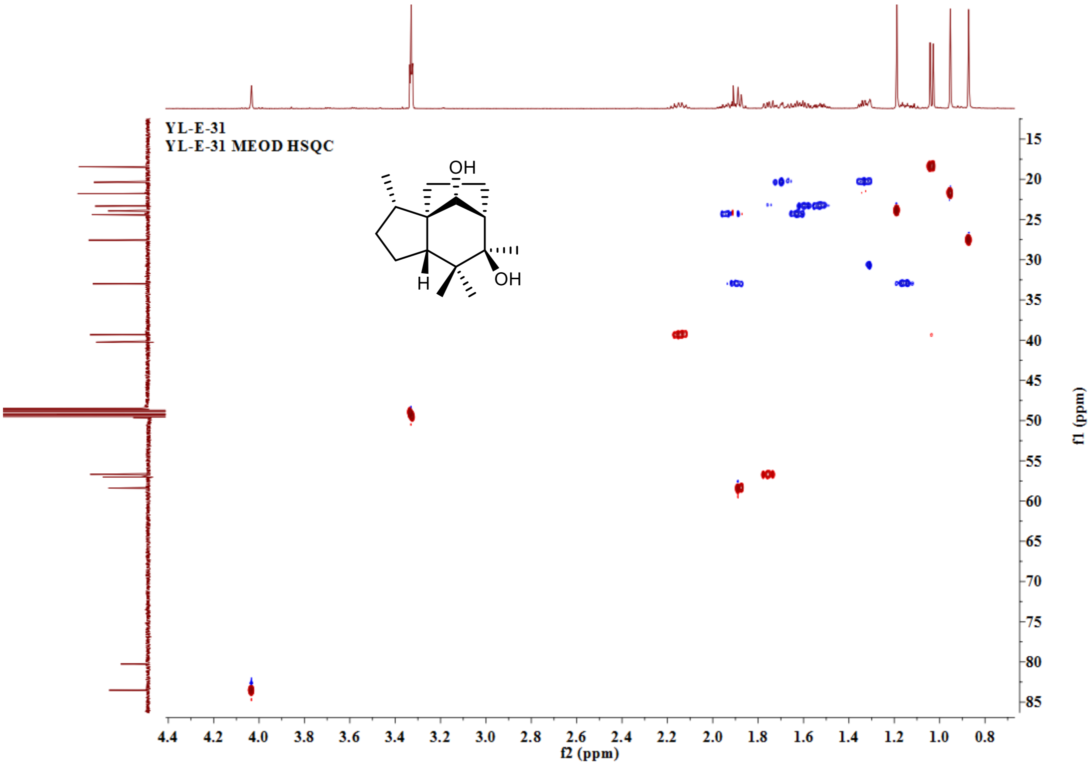

Figure S20. HSQC spectrum of compound **3** in CD_3OD

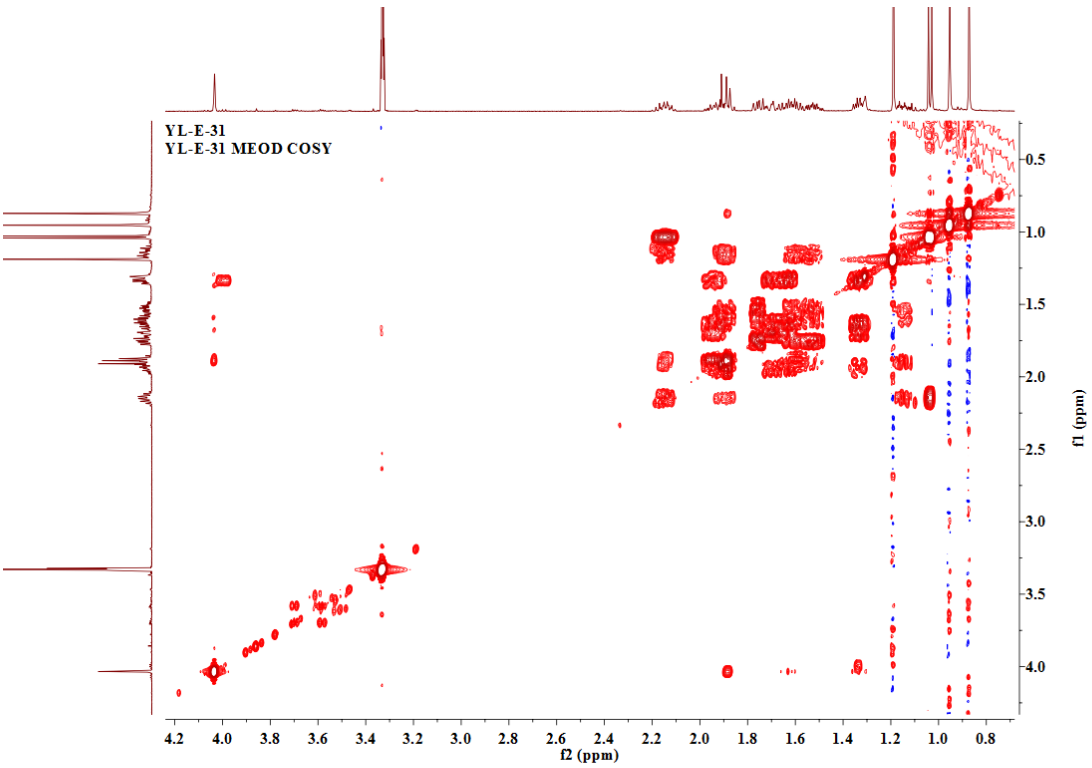

Figure S21. ^1H-^1H COSY spectrum of compound **3** in CD_3OD

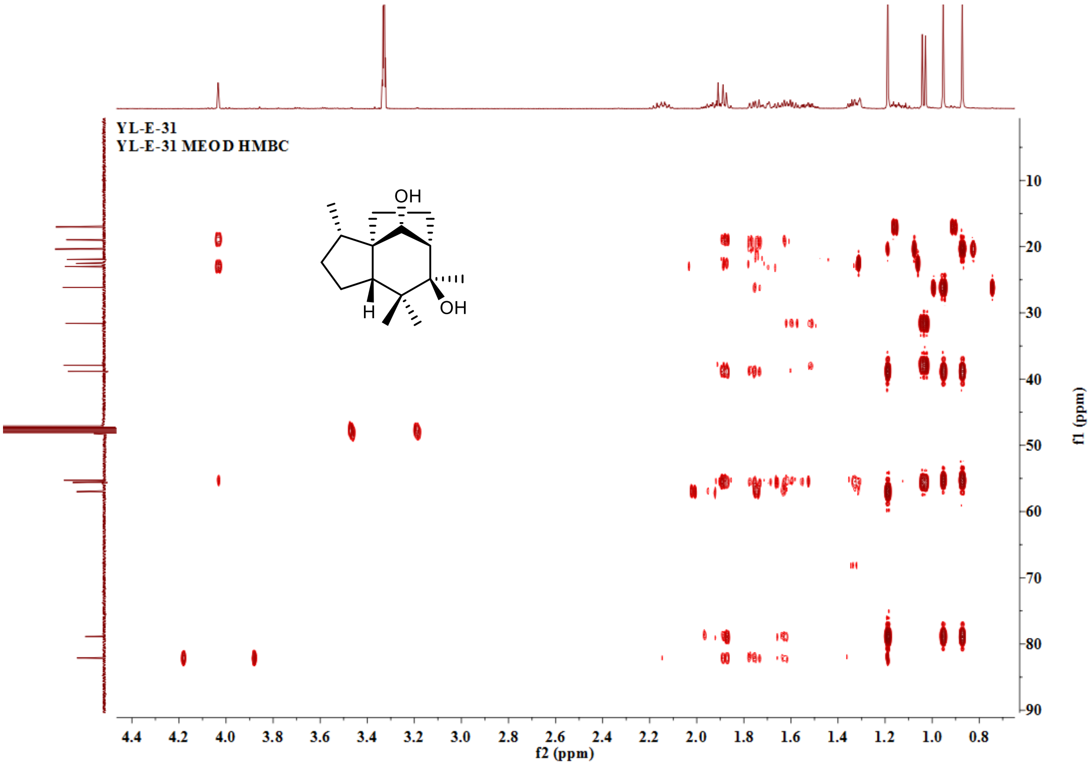

Figure S22. HMBC spectrum of compound **3** in CD_3OD

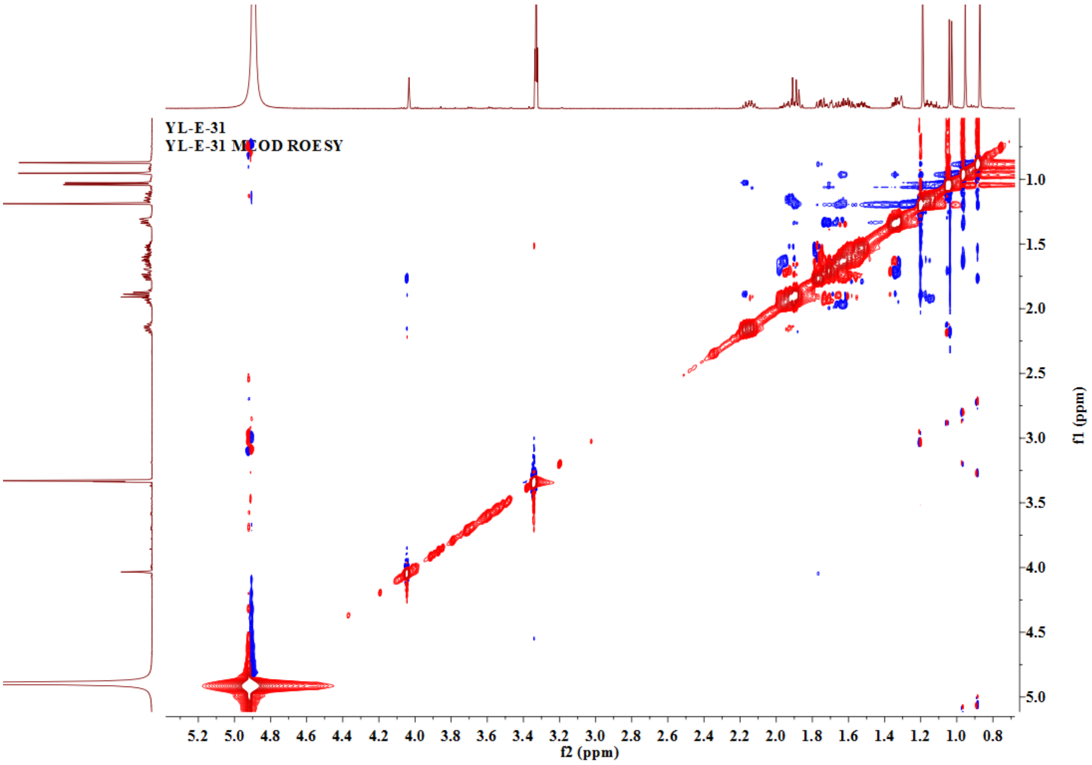

Figure S23. ROESY spectrum of compound **3** in CD_3OD

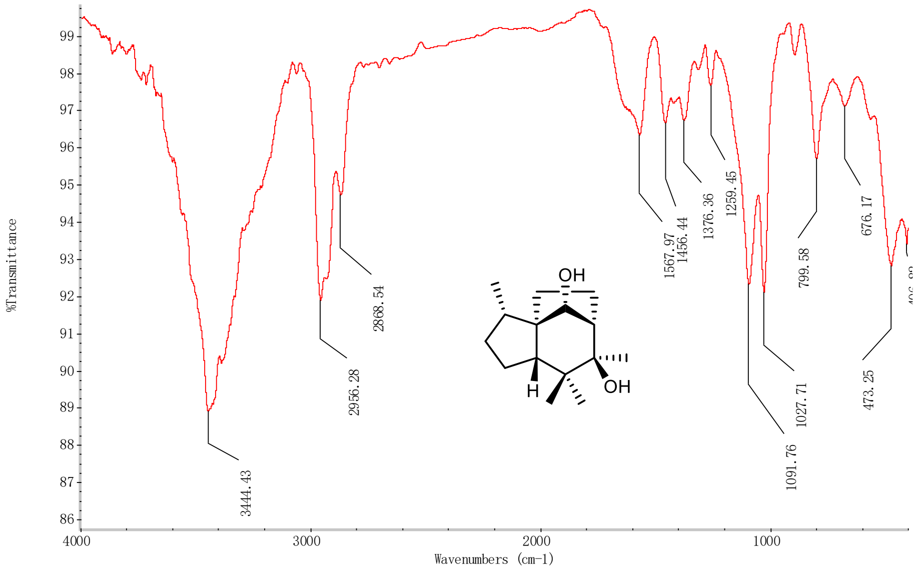

Figure S24. IR spectrum of compound **3**

Mass Spectrum SmartFormula Report

Analysis Info

Analysis Name D:\Data\A501\YJZ\2018.09.30\E-31.d
Method DirectInfusion - MS - positive.m
Sample Name E-31
Comment

Acquisition Date 2018-09-30 15:57:10
Operator Demo User
Instrument compact 8255754.20156

Acquisition Parameter

Source Type	ESI	Ion Polarity	Positive	Set Nebulizer	0.4 Bar
Focus	Not active	Set Capillary	4500 V	Set Dry Heater	180 °C
Scan Begin	50 m/z	Set End Plate Offset	-500 V	Set Dry Gas	4.0 l/min
Scan End	1300 m/z	Set Charging Voltage	2000 V	Set Divert Valve	Source
		Set Corona	0 nA	Set APCI Heater	0 °C

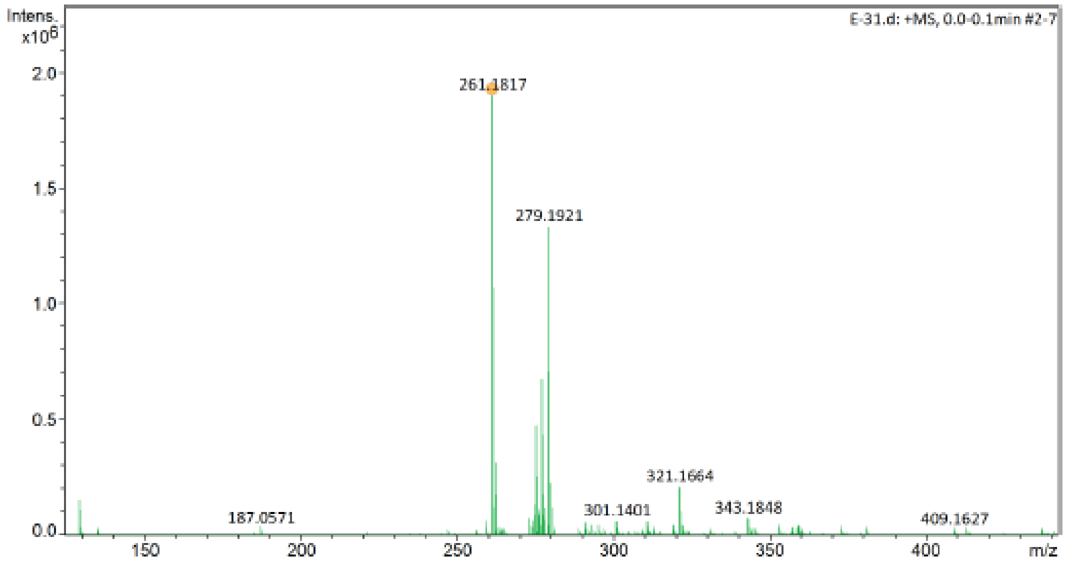

Sum Formula	Ion Formula	Meas. m/z	m/z	err [mDa]	err [ppm]
C15H26O2	C15H26NaO2	261.1817	261.1825	0.8	3.2

Figure S25. HRESIMS of compound **3**

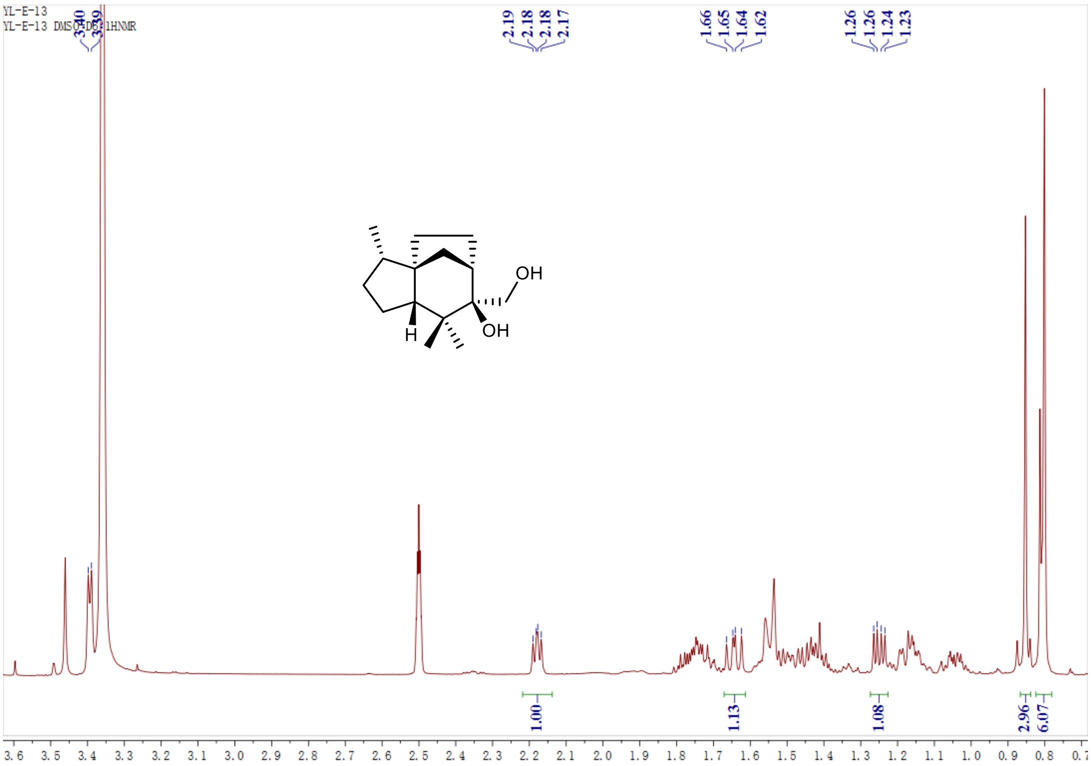

Figure S26. ^{1}H NMR (500 MHz) spectrum of compound **4** in DMSO-d_6

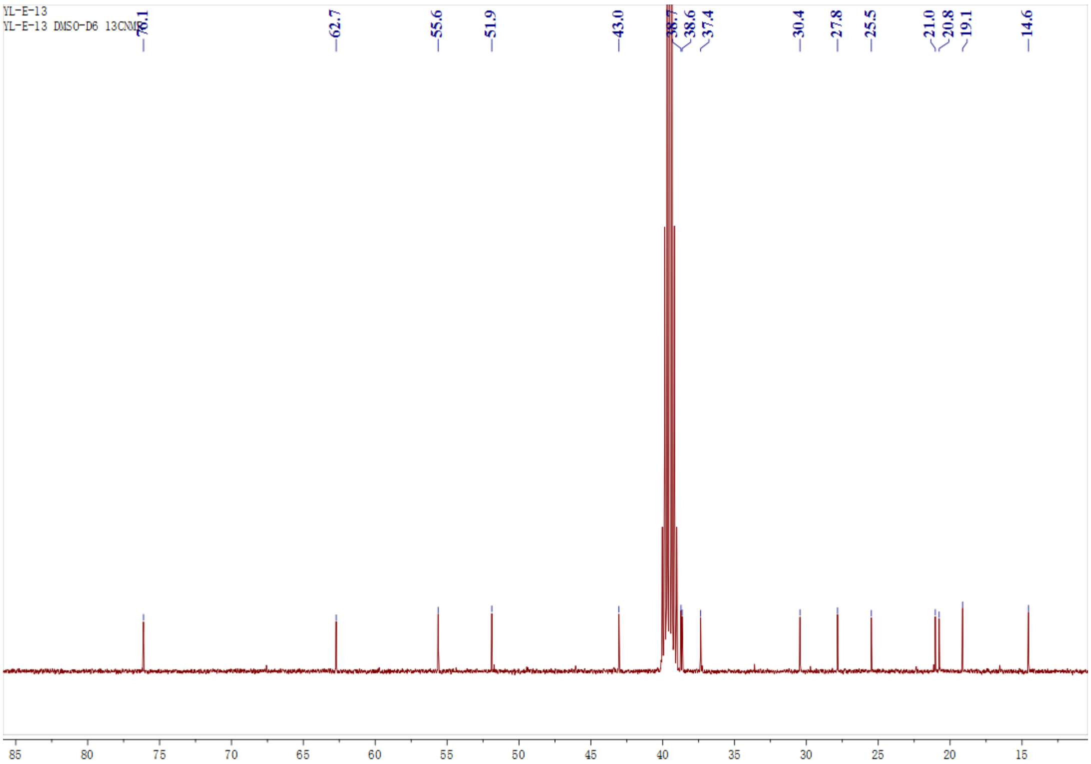

Figure S27. ^{13}C NMR (125 MHz) spectrum of compound **4** in DMSO-d_6

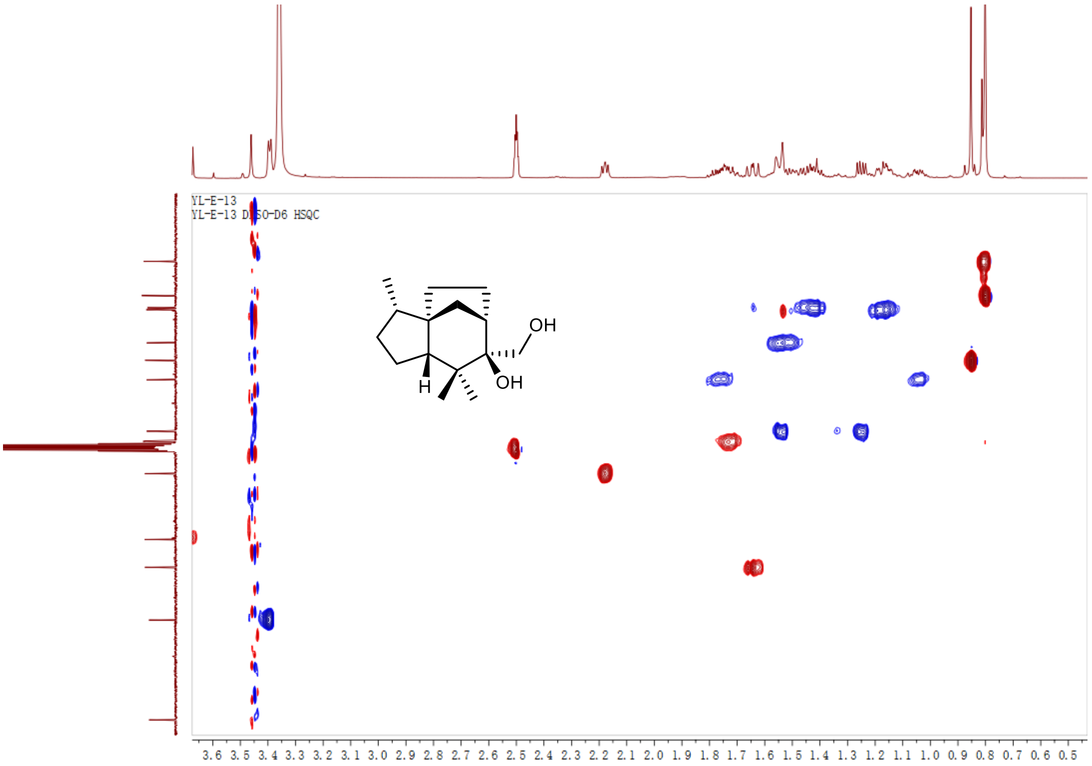

Figure S28. HSQC spectrum of compound **4** in DMSO-d_6

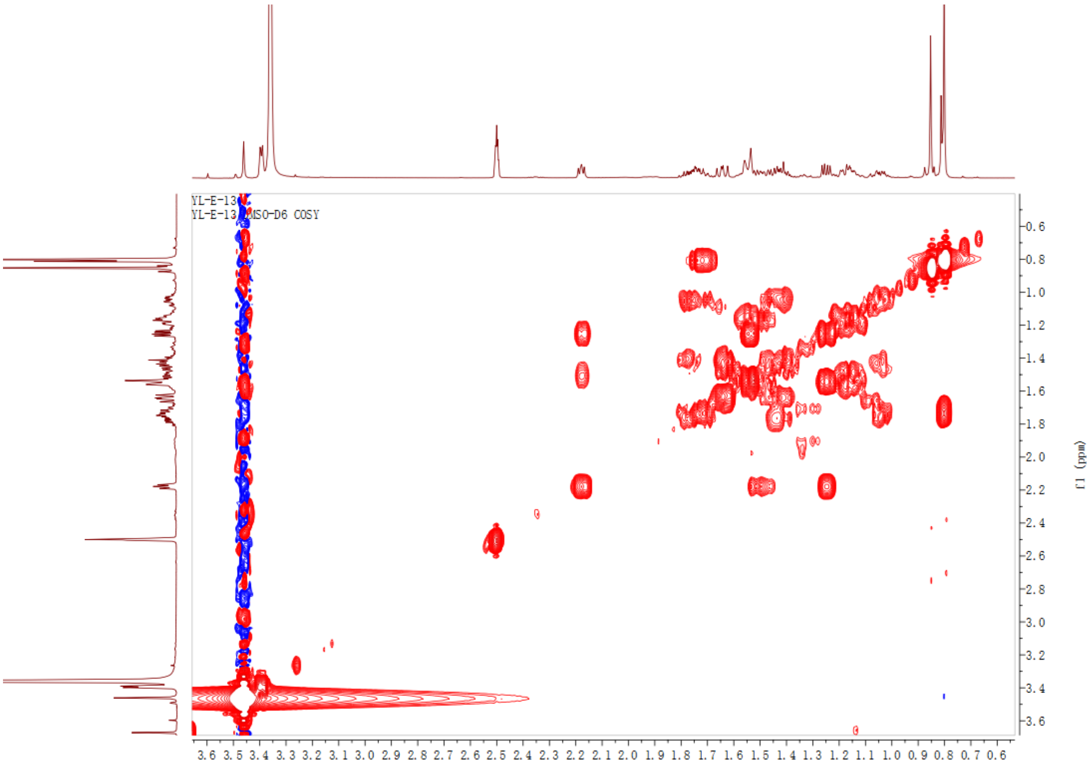

Figure S29. ^{1}H-^{1}H COSY spectrum of compound **4** in DMSO-d_6

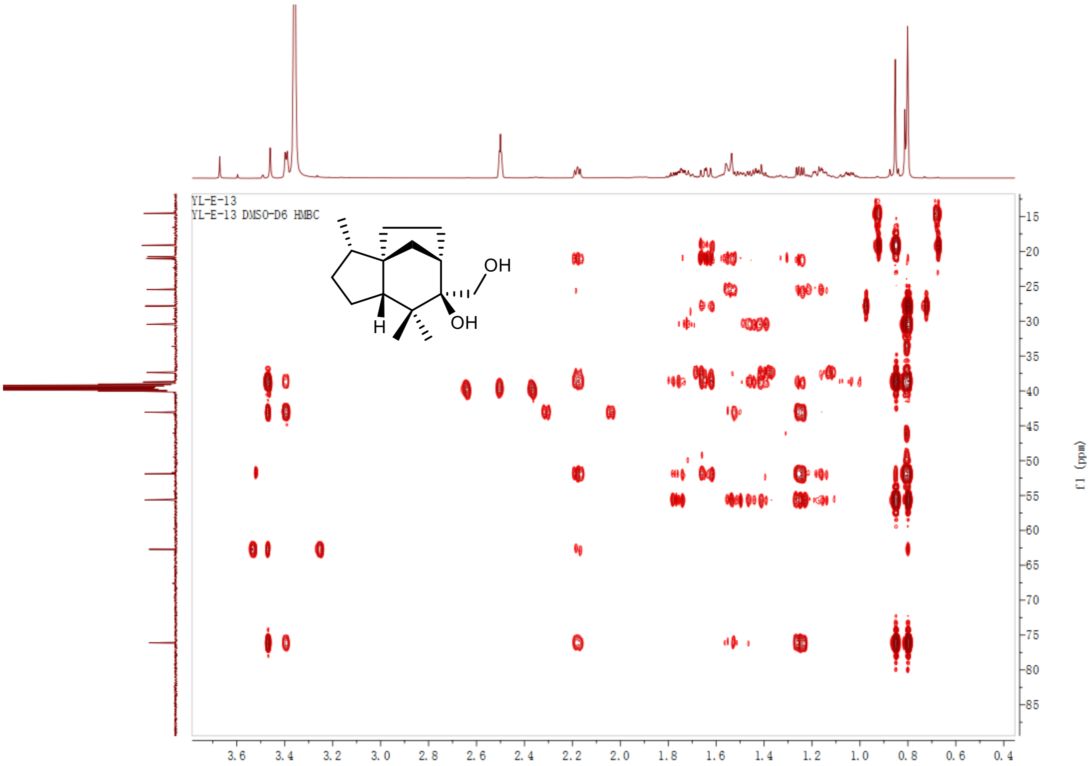

Figure S30. HMBC spectrum of compound **4** in DMSO-d_6

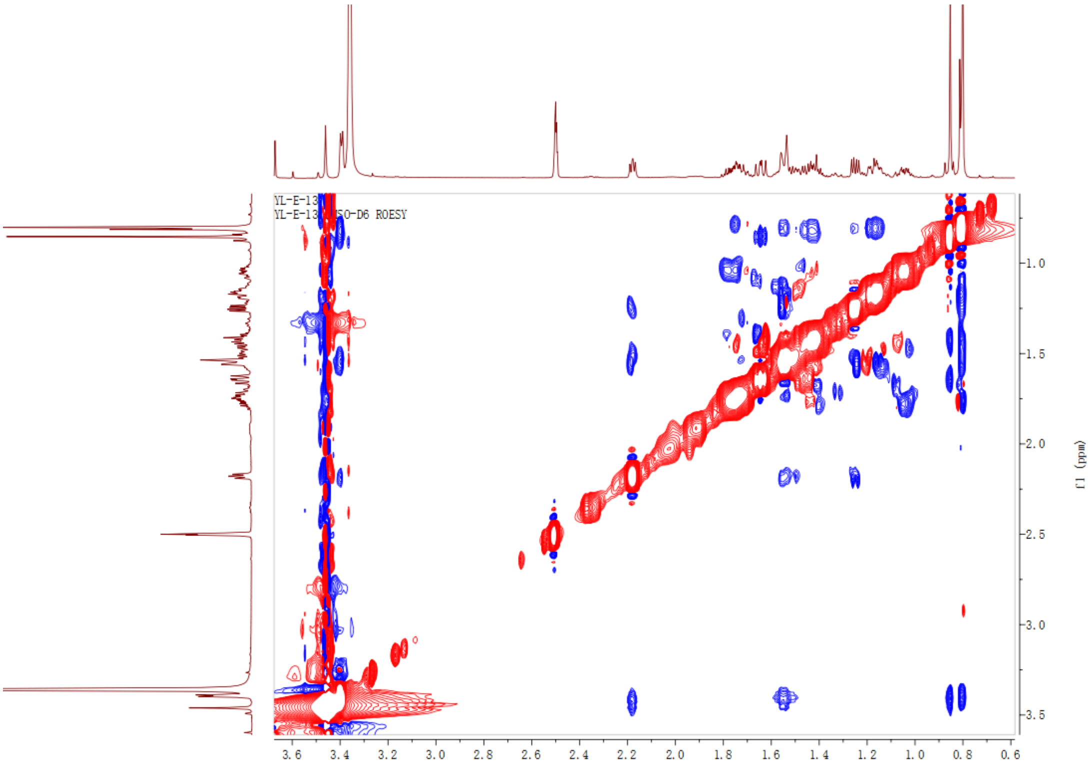

Figure S31. ROESY spectrum of compound **4** in DMSO-d_6

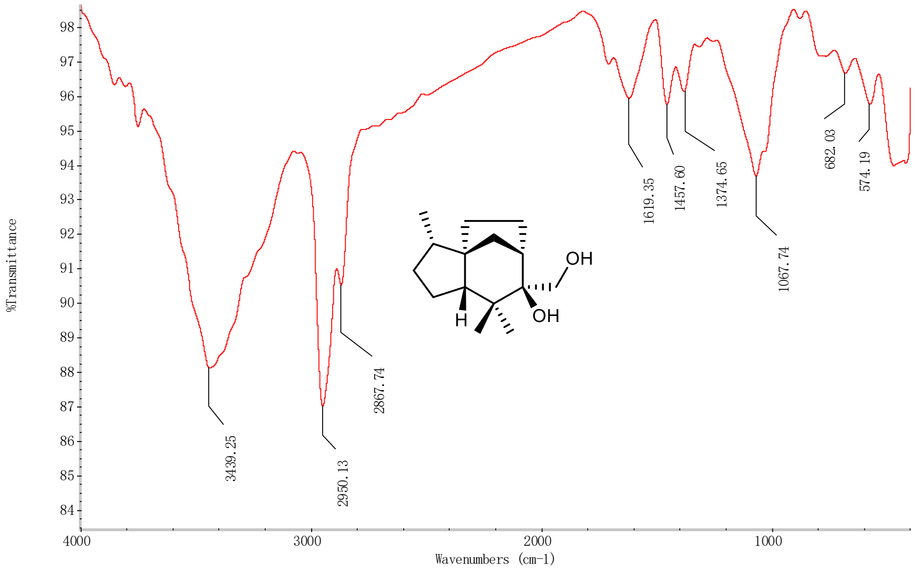

Figure S32. IR spectrum of compound **4**

Mass Spectrum SmartFormula Report

**Analysis Info**		Acquisition Date	2018-09-30 16:08:28	
Analysis Name	D:\Data\A501\YJZ\2018.09.30\E-13.d			
Method	DirectInfusion - MS - positive.m	Operator	Demo User	
Sample Name	E-13	Instrument	compact	8255754.20156
Comment				

Acquisition Parameter

Source Type	ESI	Ion Polarity	Positive	Set Nebulizer	0.4 Bar
Focus	Not active	Set Capillary	4500 V	Set Dry Heater	180 °C
Scan Begin	50 m/z	Set End Plate Offset	-500 V	Set Dry Gas	4.0 l/min
Scan End	1300 m/z	Set Charging Voltage	2000 V	Set Divert Valve	Source
		Set Corona	0 nA	Set APCI Heater	0 °C

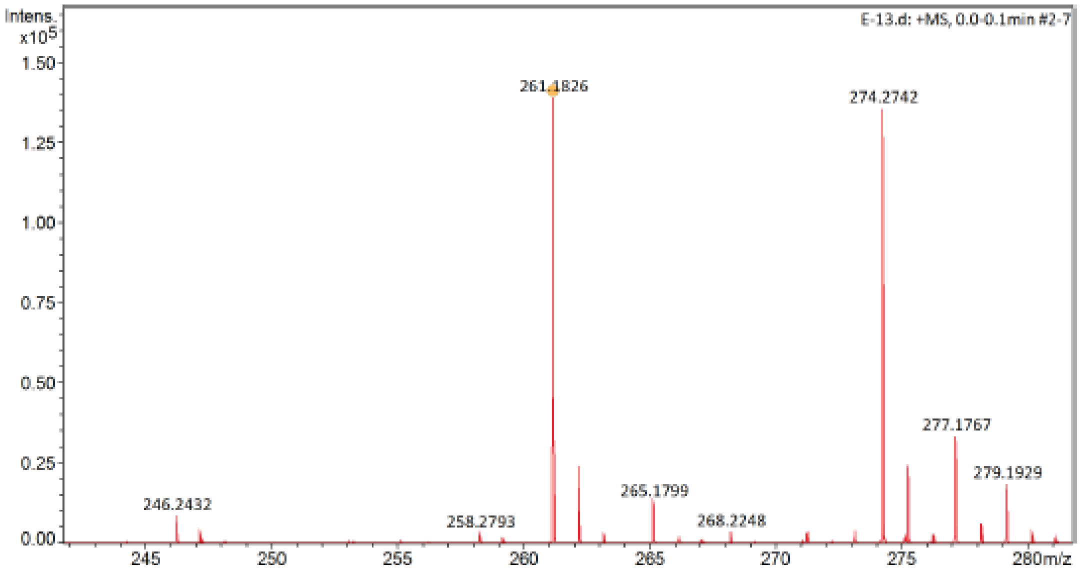

Sum Formula	Ion Formula	Meas. m/z	m/z	err [mDa]	err [ppm]
C15H26O2	C15H26NaO2	261.1826	261.1825	-0.1	-0.2

Figure S33. HRESIMS of compound **4**

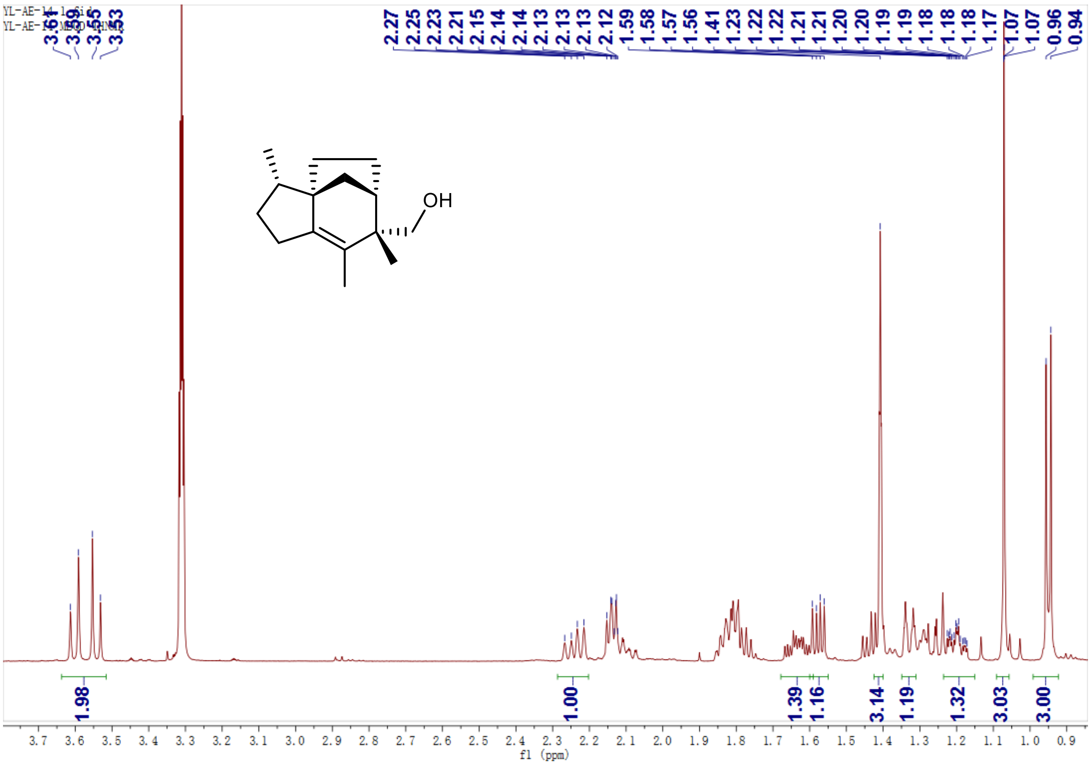

Figure S34. ^{1}H NMR (500 MHz) spectrum of compound **5** in CD_3OD

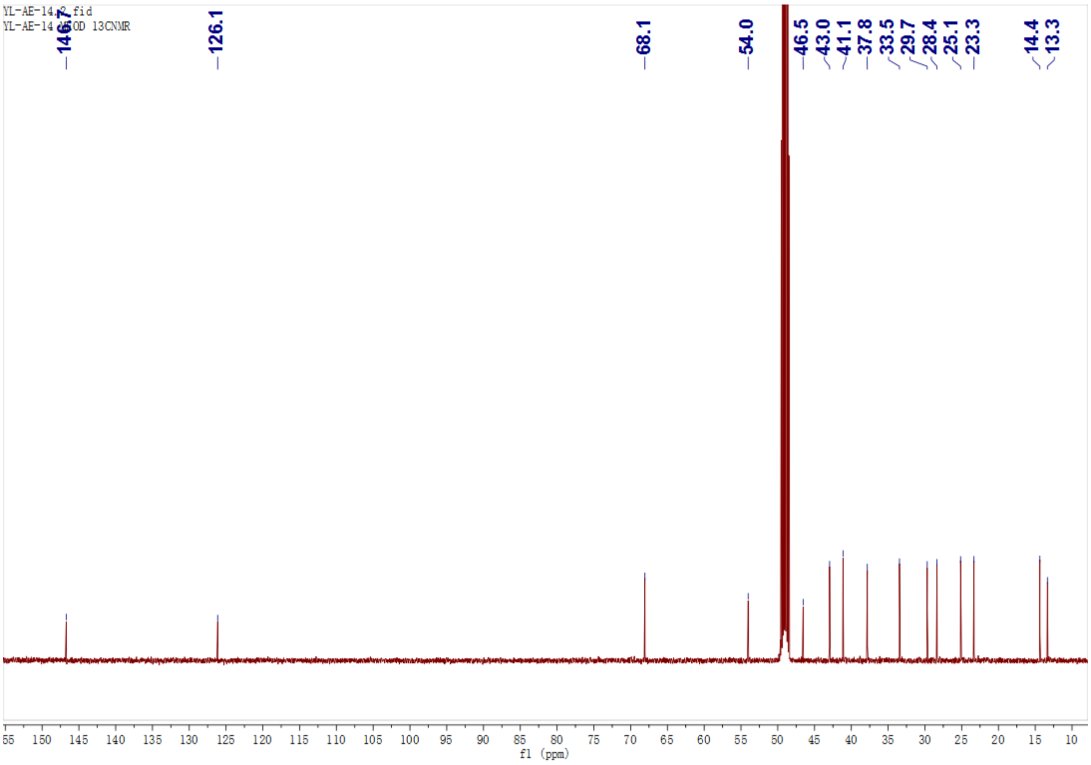

Figure S35. ^{13}C NMR (125 MHz) spectrum of compound **5** in CD_3OD

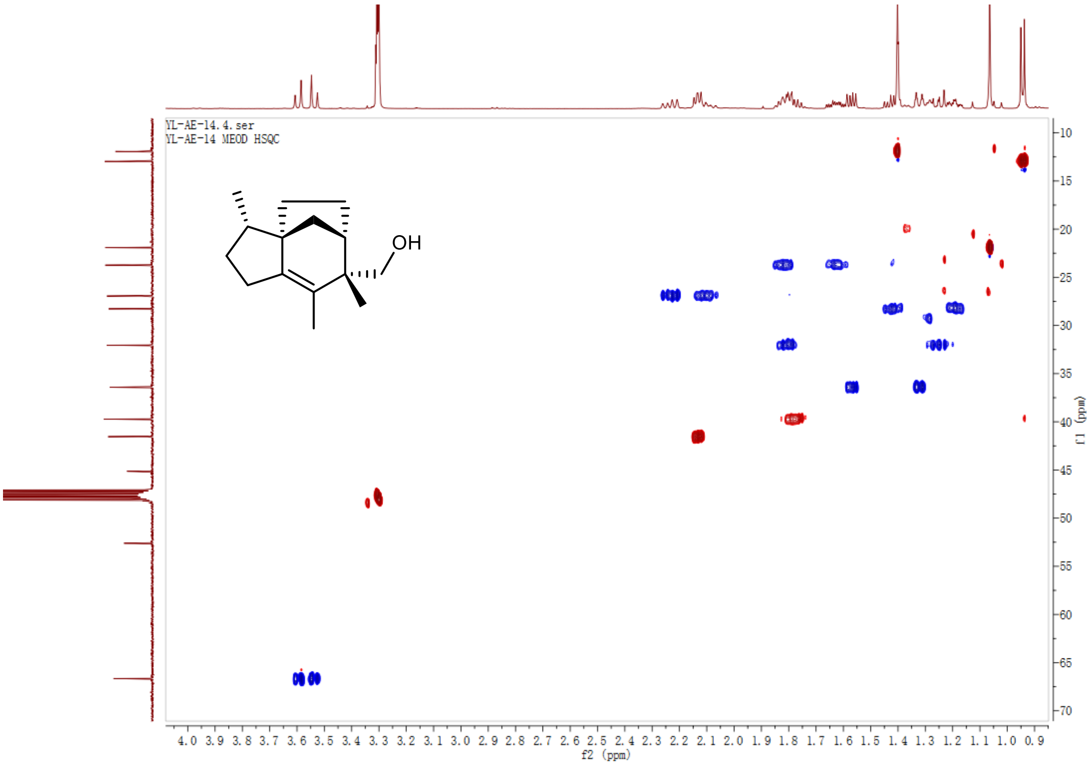

Figure S36. HSQC spectrum of compound **5** in CD_3OD

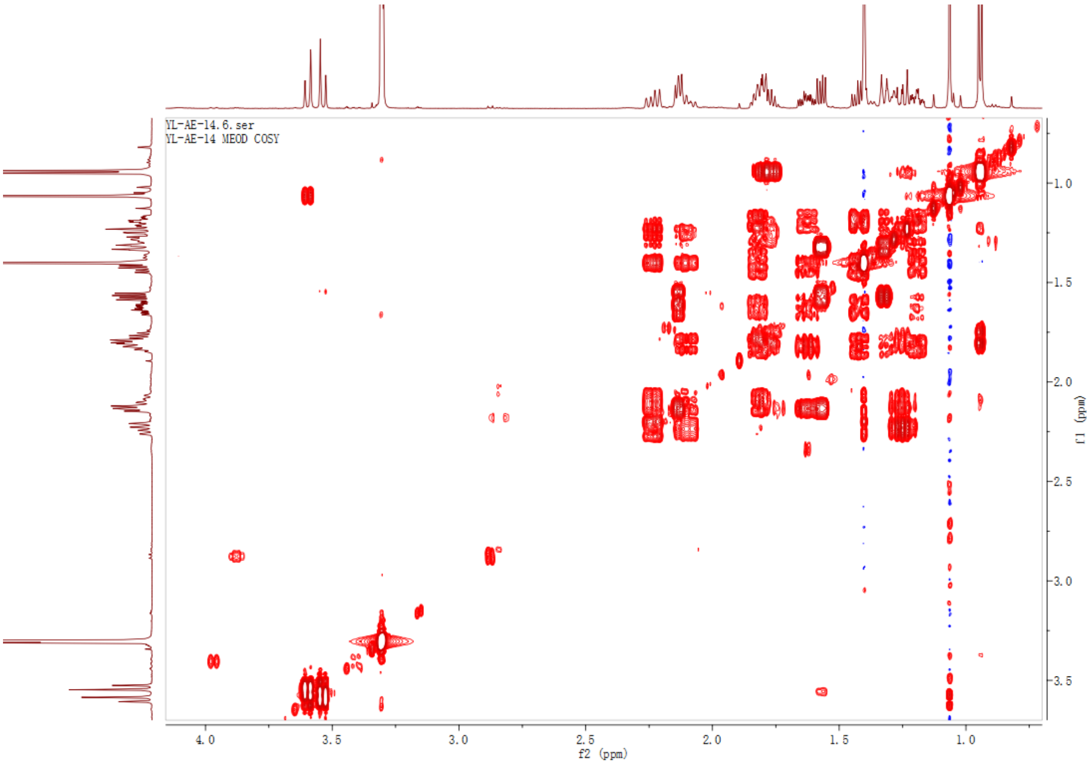

Figure S37. ^{1}H-^{1}H COSY spectrum of compound **5** in CD_3OD

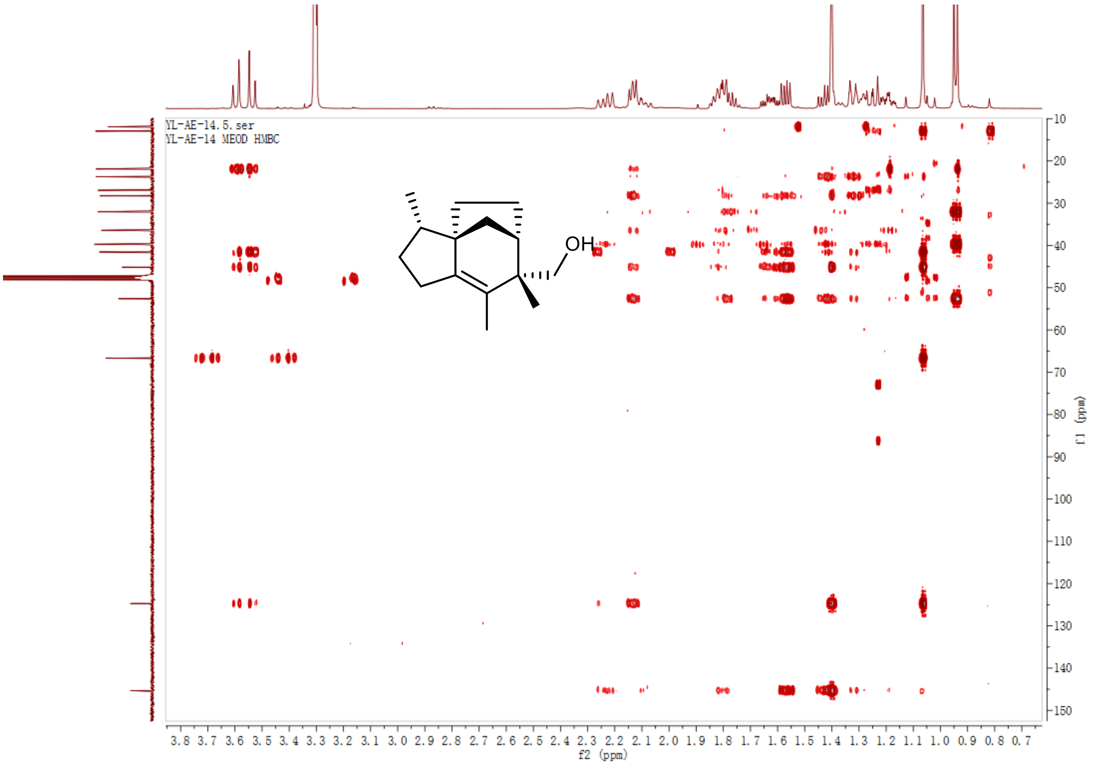

Figure S38. HMBC spectrum of compound **5** in CD_3OD

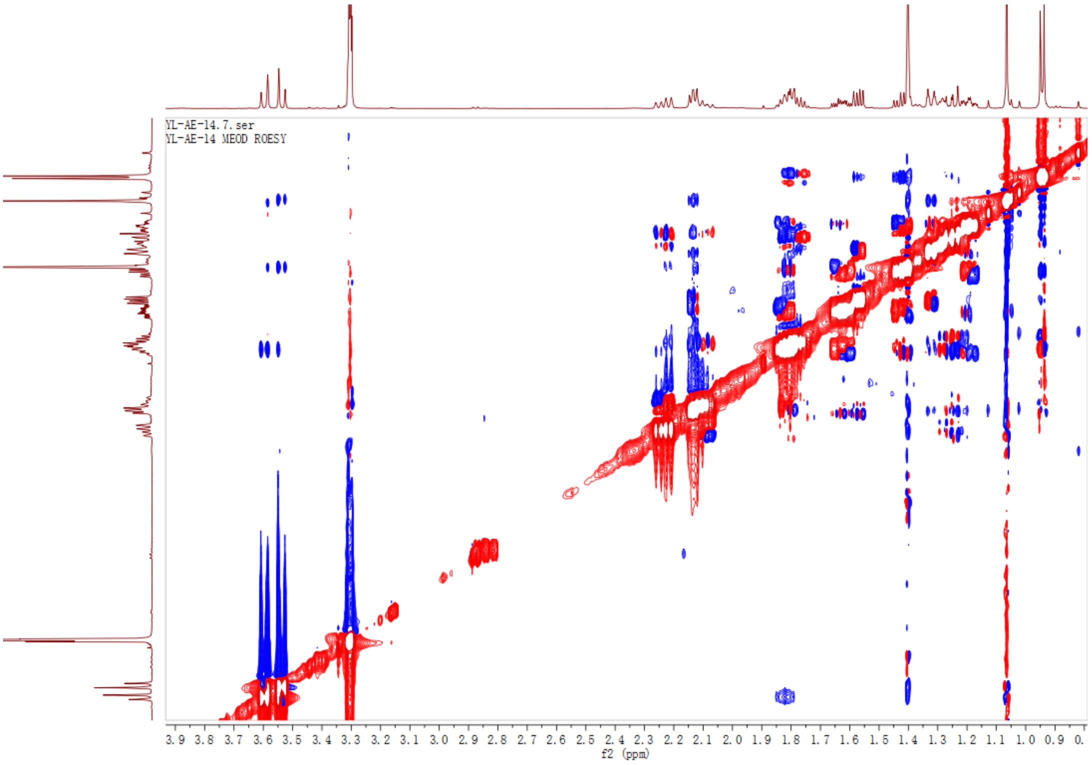

Figure S39. ROESY spectrum of compound **5** in CD_3OD

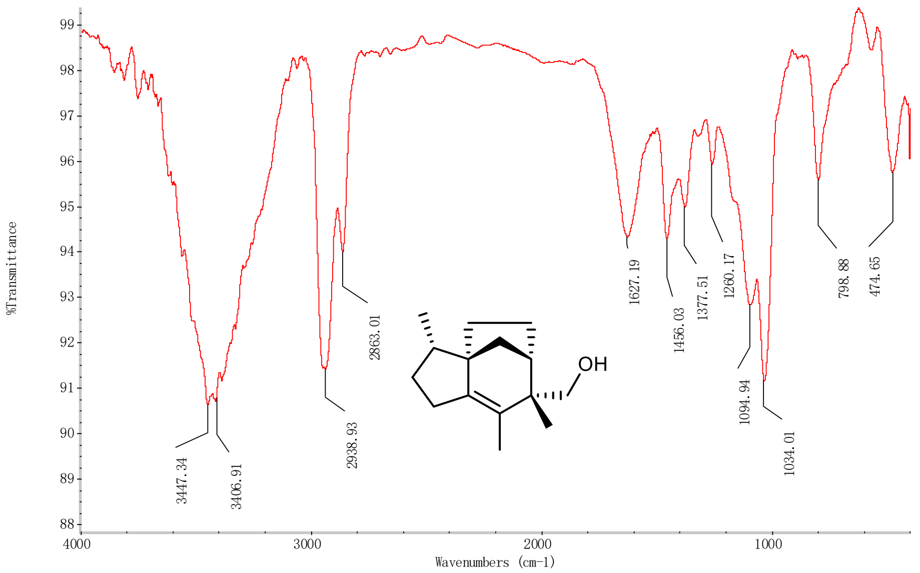

Figure S40. IR spectrum of compound **5**

Mass Spectrum SmartFormula Report

Analysis Info

Analysis Name	D:\Data\A501\YJZ\2018.09.04\YL-AE-14.d	Acquisition Date	2018-09-04 16:43:57	
Method	DirectInfusion - MS - positive.m	Operator	Demo User	
Sample Name	YL-AE-14	Instrument	compact	8255754.20156
Comment				

Acquisition Parameter

Source Type	ESI	Ion Polarity	Positive	Set Nebulizer	0.4 Bar
Focus	Not active	Set Capillary	4500 V	Set Dry Heater	180 °C
Scan Begin	50 m/z	Set End Plate Offset	-500 V	Set Dry Gas	4.0 l/min
Scan End	1300 m/z	Set Charging Voltage	2000 V	Set Divert Valve	Source
		Set Corona	0 nA	Set APCI Heater	0 °C

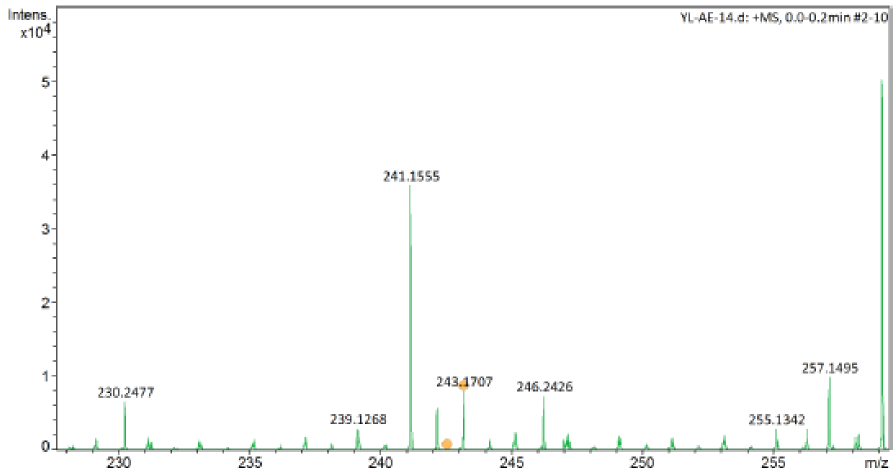

Sum Formula	Ion Formula	Meas. m/z	m/z	err [mDa]	err [ppm]
C15H24O	C15H24NaO	243.1707	243.1719	1.2	5.0

Figure S41. HRESIMS of compound **5**

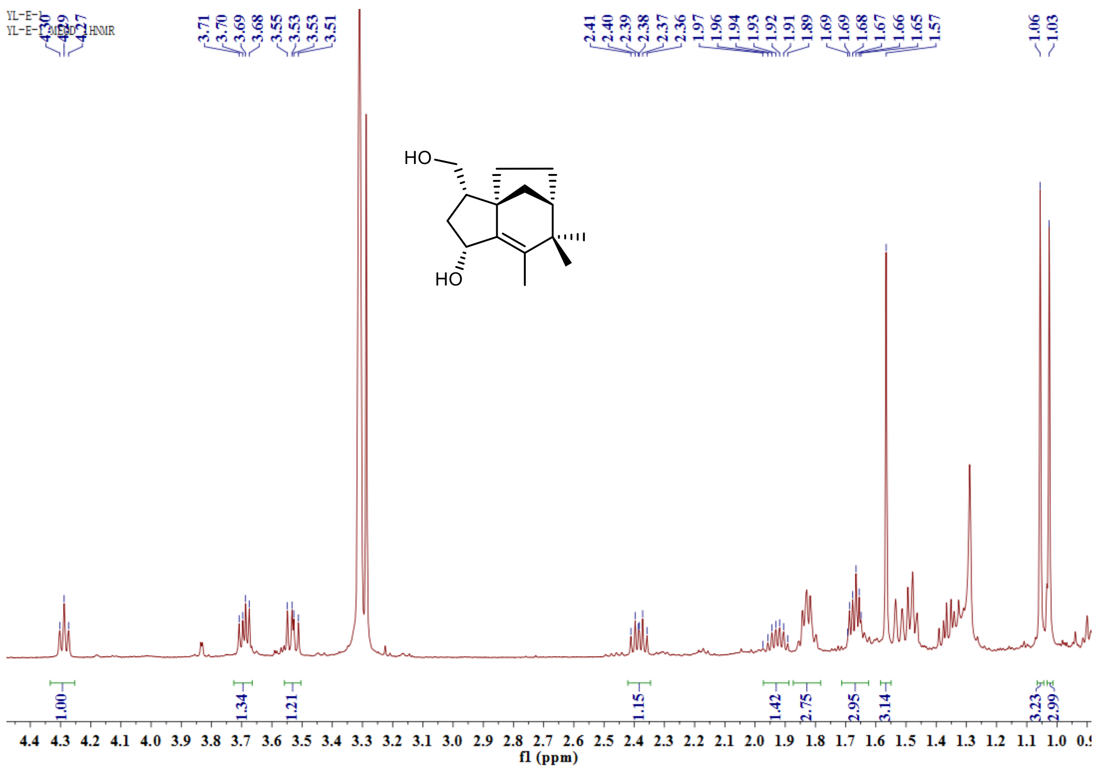

Figure S42. ^{1}H NMR (500 MHz) spectrum of compound **6** in CD_3OD

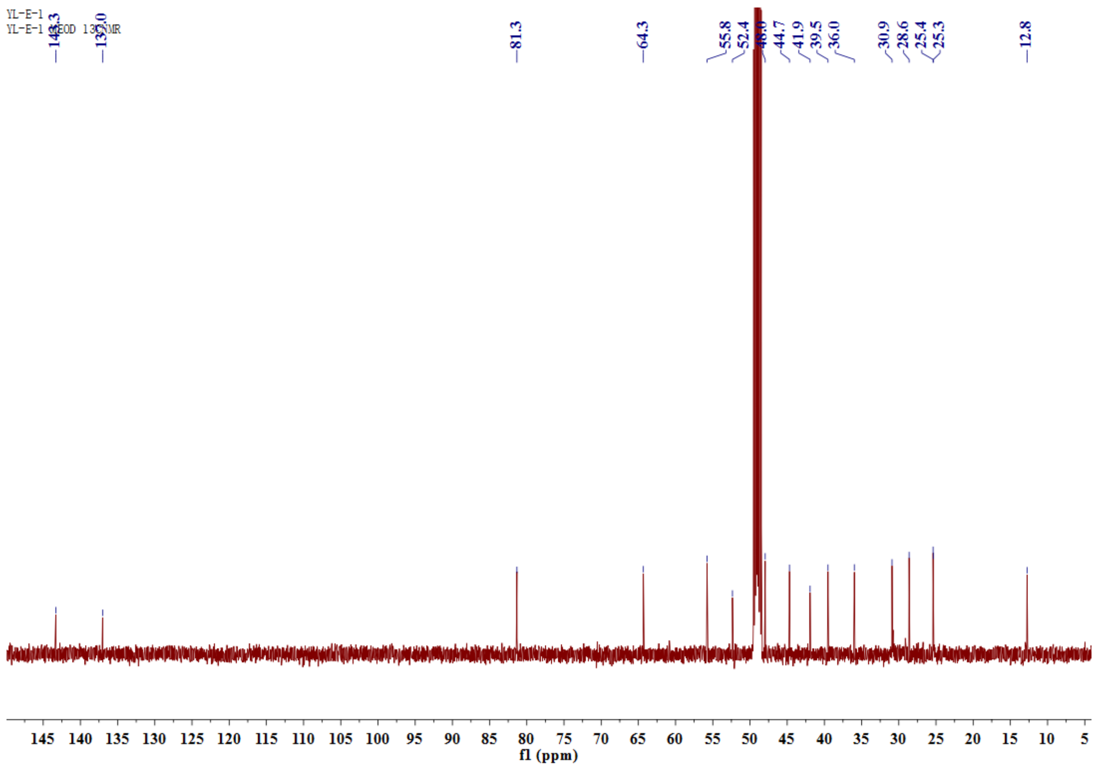

Figure S43. ^{13}C NMR (125 MHz) spectrum of compound **6** in CD_3OD

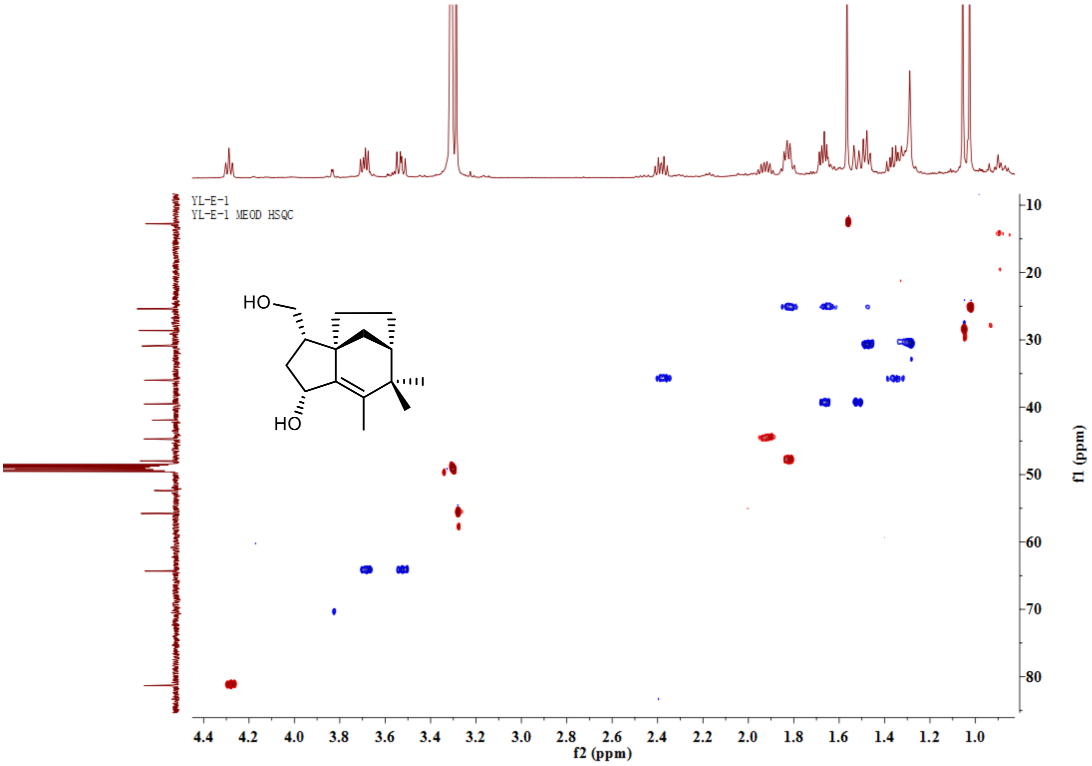

Figure S44. HSQC spectrum of compound **6** in CD_3OD

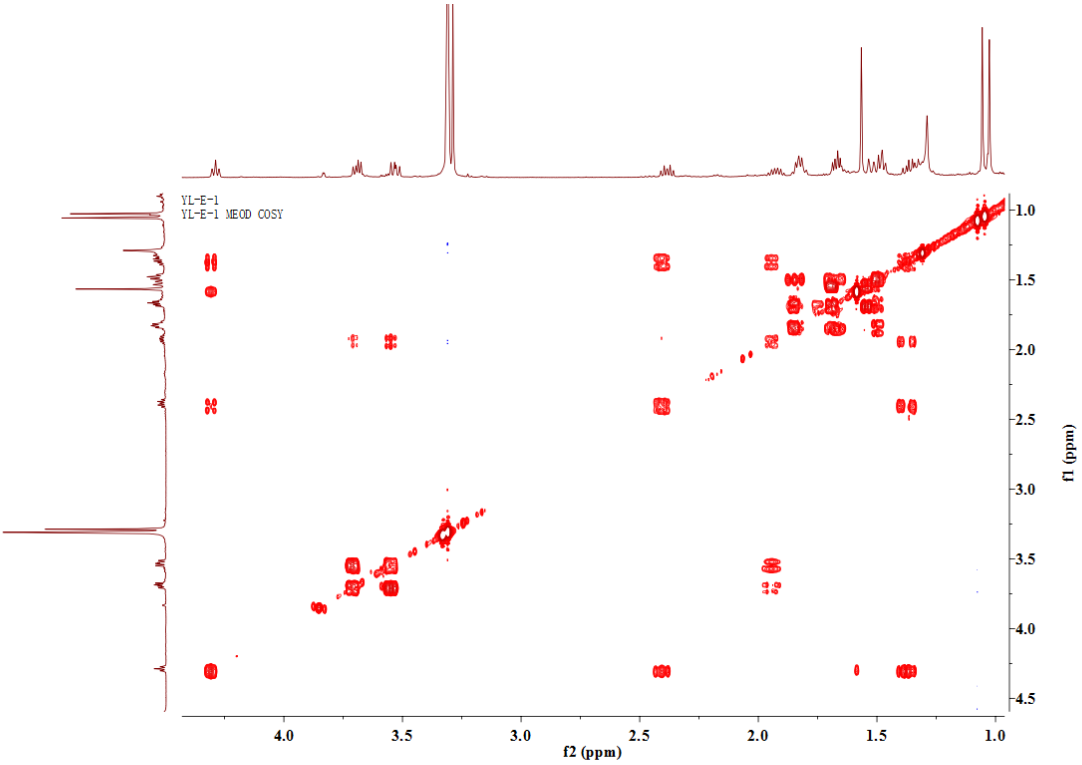

Figure S45. ^1H-^1H COSY spectrum of compound **6** in CD_3OD

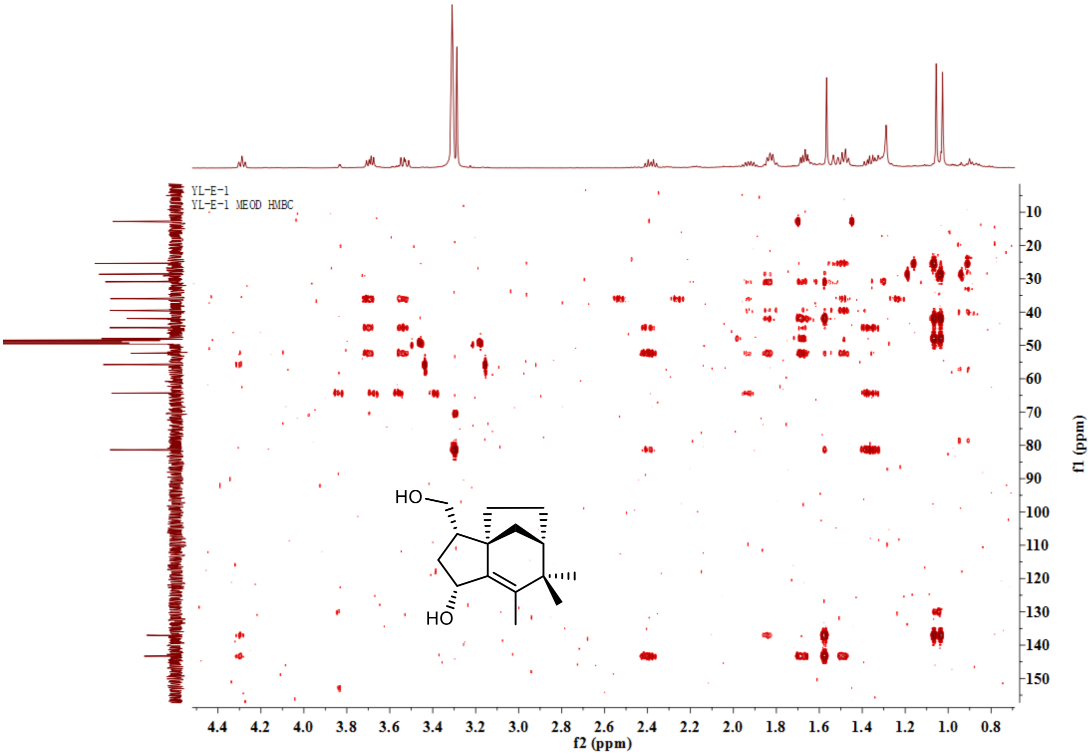

Figure S46. HMBC spectrum of compound **6** in CD_3OD

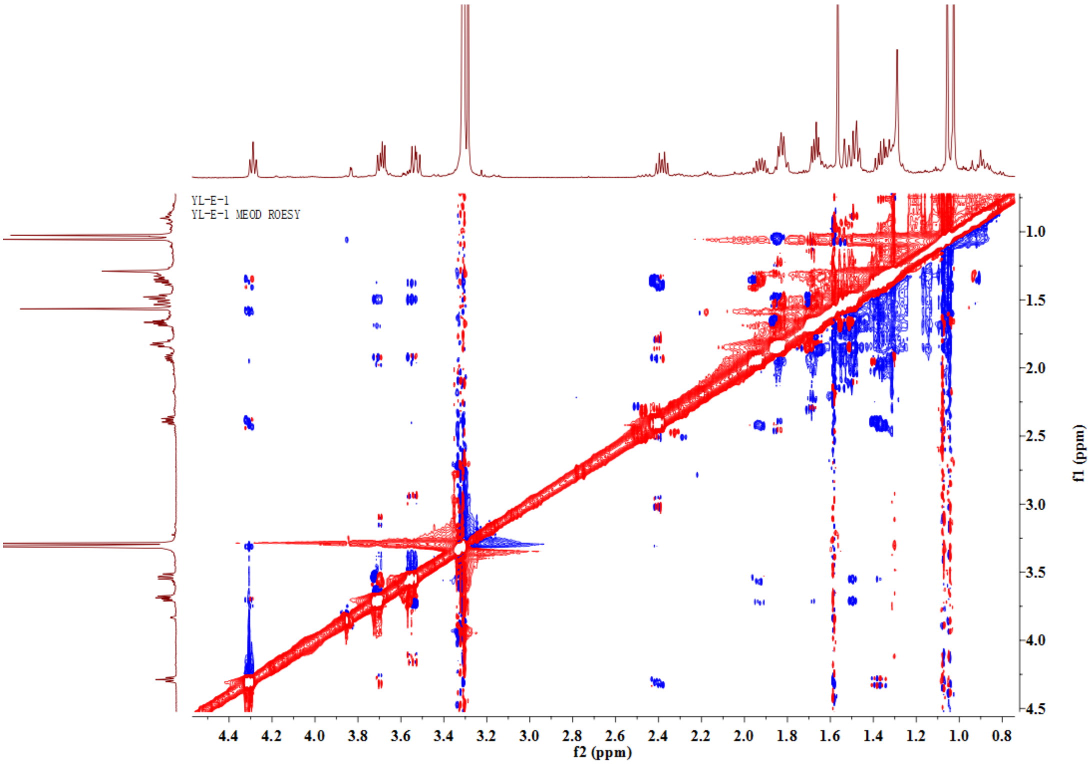

Figure S47. ROESY spectrum of compound **6** in CD_3OD

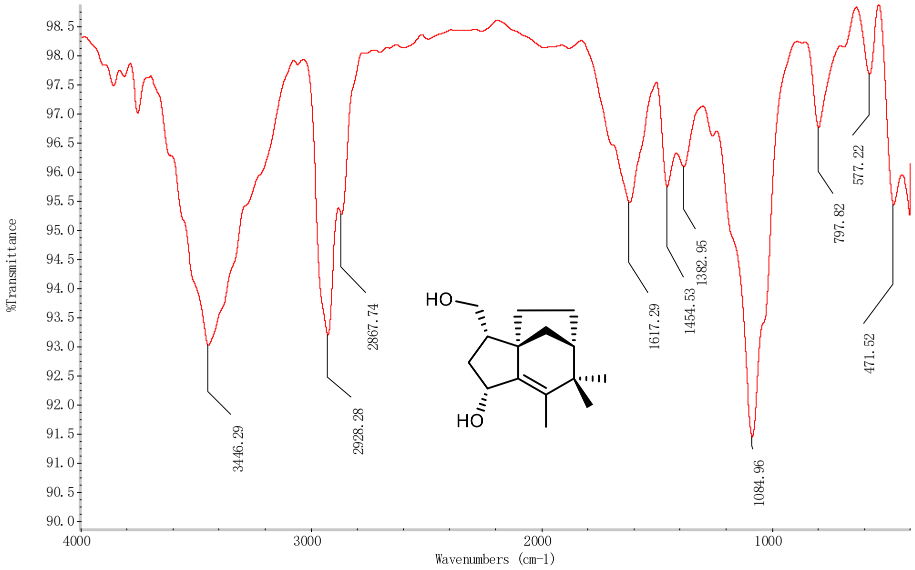

Figure S48. IR spectrum of compound **6**

Mass Spectrum SmartFormula Report

**Analysis Info**		Acquisition Date	2018-09-05 17:06:30	
Analysis Name	D:\Data\A501\YJZ\2018.09.04\YL-E-1.d			
Method	DirectInfusion - MS - positive.m	Operator	Demo User	
Sample Name	YL-E-1	Instrument	compact	8255754.20156
Comment				

Acquisition Parameter

Source Type	ESI	Ion Polarity	Positive	Set Nebulizer	0.4 Bar
Focus	Not active	Set Capillary	4500 V	Set Dry Heater	180 °C
Scan Begin	50 m/z	Set End Plate Offset	-500 V	Set Dry Gas	4.0 l/min
Scan End	1300 m/z	Set Charging Voltage	2000 V	Set Divert Valve	Source
		Set Corona	0 nA	Set APCI Heater	0 °C

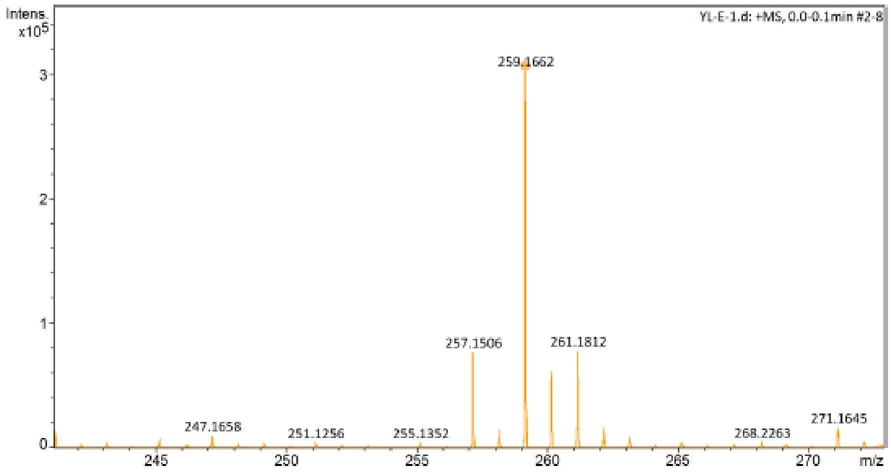

Sum Formula	Ion Formula	Meas. m/z	m/z	err [mDa]	err [ppm]
C15H24O2	C15H24NaO2	259.1662	259.1669	0.6	2.4

Figure S49. HRESIMS of compound **6**
